# Modal analysis and optimization of swimming active filaments

**DOI:** 10.1098/rsta.2024.0256

**Published:** 2025-09-11

**Authors:** John Severn, Eric Lauga

**Affiliations:** ^1^ Department of Applied Mathematics and Theoretical Physics, University of Cambridge, Cambridge, UK

**Keywords:** fluid dynamics, spermatozoa, microswimmers, elastohydrodynamics

## Abstract

Active flexible filaments form the classical continuum framework for modelling the locomotion of spermatozoa and algae driven by the periodic oscillation of flagella. This framework also applies to the locomotion of various artificial swimmers. Classical studies have quantified the relationship between internal forcing (localized or distributed internal moments or forces) and external output (filament shape and swimming speed). In this paper, we pose locomotion as a mathematical optimization problem and demonstrate that the swimming of an isolated active filament can be accurately described and optimized using a small number of eigenmodes, significantly reducing computational complexity. In particular, we reveal that the motion of a filament with monophasic forcing, relevant to recently proposed artificial swimmers, is governed by exactly four forcing eigenmodes, only two of which are independent. We further present optimizations of such swimmers under various constraints.

This article is part of the theme issue ‘Biological fluid dynamics: emerging directions’.

## 1. Introduction

Microorganisms employ a variety of mechanisms to self-propel through viscous fluids [1–6]. Due to their small size, their swimming is governed by low-Reynolds-number hydrodynamics, i.e. Stokes flows [7], which is the assumed flow regime for the swimmers (whether micro-scale, millimetre-scale or larger) discussed in this paper. In this regime, inertial effects in the fluid become negligible relative to viscous effects, and the locomotion kinematics are constrained by the scallop theorem: time-reversible motion of the swimmer’s body cannot produce any net swimming [8] (so named because a small scallop that repeatedly opens and closes would not be able to swim in the Stokes flow limit). Consequently, microorganisms must employ non-time-reversible actuation to undergo net locomotion. Such mechanisms include the rotation of rigid helical flagellar filaments of bacteria, such as those employed by the model organism *Escherichia coli*, that rotate to push the swimmer forwards [2], or the waving dynamics of the two flexible flagella of the green algae *Chlamydomonas* that act like arms to pull the swimmer forwards [3]. Perhaps the most commonly known method of propulsion is that of spermatozoa, which utilizes an undulating flexible flagellum to transmit travelling waves that push fluid backwards and hence push the swimmer forwards [4,5].

It has long been known that the actuation of the spermatozoa flagellum is not simply localized to the point of attachment with the cell body, but is instead continuously distributed along the entire flagellum length [9–12]. This is facilitated by the axoneme, the internal structure of the flagellum, containing molecular motors that power the relative sliding of microtubules [13,14], leading to an effective (and sophisticated) distribution of internal moments and forces that are functions of both space (location along the flagellum) and time [4]. Not only does understanding the swimming dynamics of spermatozoa have fundamental interest for cellular biology and fertility science [15], but the simple form of the spermatozoa model (a single flexible filament attached to a passive body) makes it an ideal basis for theoretical studies in mathematical biology [5,16–21], and for the design and fabrication of experimental, artificial swimmers [22,23], including those with biomedical applications [24].

While we aim to make the analysis in this paper as general as possible, we keep in mind two intuitive and motivational examples of such sperm-like artificial swimmers. Dreyfus *et al.* [25] constructed a micro-swimmer with a flagellum made of connected magnetic beads, actuated by an oscillating external magnetic field, that propelled a payload in the form of a red blood cell. This design was refined in later works to produce increasingly effective and diverse swimmers [26–29]. However, such swimmers are not truly self-propelled, relying on external magnetic fields. In a creative solution to this limitation, Williams *et al.* [30] created a polymeric sperm-like millimetre-scale swimmer that was powered by heart muscle cells (cardiomyocytes) cultured onto the side of the flagellum, and this concept of muscle-powered swimmers has since then been diversely explored [31–33]. We later see that the magnetic swimmer [25] and the biohybrid swimmer [30] provide key and intuitive examples for the application of the models developed in this paper.

From a fundamental physical point of view, flexible flagella are subject to three principal forces: viscous drag from the surrounding fluid, active internal forcing from the biological activity at the axoneme level (or artificial equivalents) and passive elasticity of the flagella. Due to the lack of inertia, these forces must always instantaneously balance, producing classical partial differential equations (PDEs) which govern the dynamics of the filament [34]. These elastohydrodynamic (EHD) equations directly relate the time-varying shape of the flagella to the active internal forcing and they have been used to model both real spermatozoa [4] and sperm-like artificial swimmers [22–25,30].

Mathematically, for a given active forcing inside the flagellum, the EHD equations can be solved to determine the filament motion [4]. Then, since the total hydrodynamic forces and moments exerted by a swimming microorganisms must be zero at all times, the filament motion can be used to determine the swimming kinematics via a global force and moment balance. These calculations are generally numerical, but a fully analytical approach becomes available if perturbations are assumed to be small relative to a straight flagellum. However, to the authors’ knowledge, no single formula for the swimming speed directly in terms of the active forcing (without the need to explicitly calculate the filament motion) has yet been offered, even in this linearized limit. In addition to this linearization, proposed experimental artificial swimmers often utilize forcing that is entirely in phase [25,30], simplifying the problem further. Despite these simplifications, optimizing the configuration of the swimmer, involving parameters such as filament elasticity, fluid viscosity and active force distribution, remains a largely brute-force computational (or experimental) task [26,27,29,30].

In this paper, we consider an idealized version of a biological or artificial flagellum: an active flexible filament waving in a Stokes flow under small-amplitude forcing and with no head or cell body. In the first part of our paper, revisiting classical work, we solve the classical EHD equations and derive a new formula directly linking the active forcing to the swimming speed via a symmetric swimming speed function, bypassing the need to explicitly calculate the filament motion. In the second part of our paper, we pose an optimization problem wherein we maximize the swimming speed subject to a fixed forcing magnitude, and show that the solutions to this optimization problem are the eigenmodes of the swimming speed function, which form an orthonormal basis for all possible forcing functions. We demonstrate optimization procedures to maximize the swimming speed of the filament subject to a variety of constraints, resulting in a reduced computational complexity compared to classical methods. We pay particular attention to the case of monophasic forcing, relevant to artificial swimmers studied experimentally [25,30]. By applying the optimization procedure to such filaments, we find, remarkably, that only four of the eigenvalues are non-zero, and analytically calculate these eigenvalues and their corresponding eigenfunctions. Two of these eigenmodes are simply reflections of the other two (their eigenvalues being negative), and one eigenvalue dominates the other under optimal physical parameter conditions. Swimming is therefore governed approximately by just a single eigenvalue and eigenmode pair. Finally, we demonstrate that analysis can be applied to this eigenmode pair to optimize swimming far more efficiently than brute-force computation.

The paper is organized as follows. In §2, we obtain the full dimensionless EHD equations for a general moment forcing function. We then linearize these equations for a small internal forcing, and solve the resultant forced hyperdiffusion equation to determine the filament shape (equations (2.29)–(2.31)) in terms of the Green’s function G (appendix A). Using this solution and global force balances, we identify the swimming speed of the filament (equation (2.35)) entirely in terms of the moment forcing function and a symmetric swimming speed function $G_{swim}$ (equation 2.36) that is constructed from G, bypassing the explicit solution for the filament shape. In §3, we next consider an eigenvalue/eigenfunction problem for $G_{swim}$ that can be solved numerically (for arbitrary forcing phases) to obtain an orthonormal eigenmode basis (equation (3.1)) from which to construct the forcing function, and evaluate the swimming speed (equation (3.2)). We also construct an optimization procedure, which shows that these eigenfunctions produce local minima or maxima for the swimming speed, subject to a fixed forcing magnitude constraint.

We next demonstrate the advantages of this method using two key examples. We first consider a travelling wave of forcing, obtaining similar results to those observed in biological spermatozoa [4], and identifying the optimal forcing wavelength and a range of near-optimal values for the dimensionless parameter Sp that denotes the relative elastic properties of the filament (figure 2*a*). We then consider the case of monophasic forcing, analytically solving for the four non-zero eigenvalues and eigenfunctions. Considering potential applications to experimental, artificial swimmers [25,30], we demonstrate analyses that can be used to optimize the swimming speed, subject to a variety of physical constraints and limitations. In particular, and somewhat counter-intuitively, we show that swimming with a fixed total forcing magnitude is optimized in the limit of single-point actuation (rather than distributed forcing) and produces a far greater swimming speed than even eigenfunction forcing under the same constraint. We conclude in §4 with a summary of the key results, and offer a discussion of potential extensions of our modal approach to more complex waving swimmers.

## 2. Classical elastohydrodynamics of active filaments

A great deal of classical work has been done in modelling the response of the filament shape to both proximal and internal forcing, and how the resultant motion induces driving forces and locomotion [1,4,5,18,34]. Here we summarize this work, in particular presenting the classical linearized, dimensionless EHD equations that balance the hydrodynamic, elastic and active internal forces. We then solve these for general active forcing using a Green’s function, and thence derive the swimming speed function $G_{swim}$. Unless stated otherwise, we work below in the lab frame with standard Cartesian (x,y) axes; this is the frame in which the fluid is stationary in the far field, and in which the filament achieves net displacement through swimming, as would be observed under a stationary microscope.

### (a) Summary of classical work

**Figure 1.**
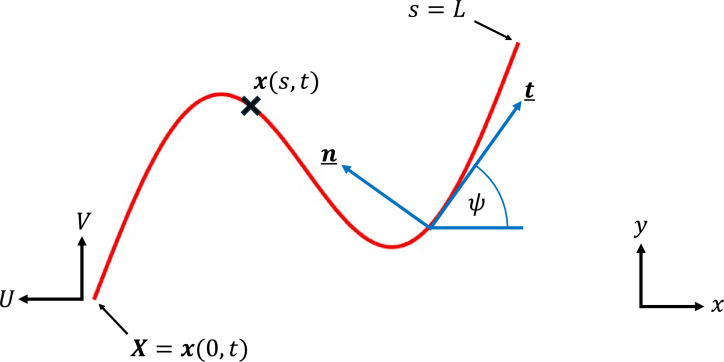
Active filament. Parameterization of a (dimensional) filament of total length L. Notation includes: arc length 0≤s≤L, tangent angle ψ, tangent vector t, normal vector n, tip position X and instantaneous tip velocity (−U,V).


*Parameterization and notation*. We begin by parameterizing the filament of length L by its arc length 0≤s≤L and tangent angle ψ(s,t)*,* giving tangent vector t=(cos⁡ψ,sin⁡ψ) and normal vector n=(−sin⁡ψ,cos⁡ψ)*,* as shown in figure 1. The front tip, s=0, has position X(t) and instantaneous velocity (−U,V) (note the sign convention applied to U; forwards swimming gives U>0). The position x(s,t) and velocity u(s,t) of a material point along the filament are then given by

(2.1)x(s,t)=X(t)+∫0s(cos⁡ψ(s′,t)sin⁡ψ(s′,t)) ds′,(2.2)u(s,t)=(−U(t)V(t))+∫0s(−sin⁡ψ(s′,t)cos⁡ψ(s′,t))ψt(s′,t) ds′.

*Dimensional hydrodynamic and EHD equations*. The filament is subject to elastic forces with bending modulus A; as is standard, A has units Nm${,}^{2}$, and is a measure of the stress required to bend the filament, see equation (2.4). The filament is also subject to an active (internal) moment forcing m(s,t)*,* interpreted physically as the moment exerted by material at $s^{+}$ on material at $s^{-}$. Therefore, $m_{s}$ is both the active moment per unit length acting on the filament, and the force exerted by material at $s^{+}$ on material at $s^{-}$. It follows that $m_{ss}$ can be interpreted as the active force per unit length acting on the filament. Fluid drag acting on the filament is calculated using resistive-force theory [35], with parallel motion (i.e. motion in the direction of the long axis of the filament) incurring a drag force per unit length of $c_{\|}$ per unit filament speed, and perpendicular motion incurring a drag force per unit length of $c_{⟂}$ per unit filament speed, giving an overall hydrodynamic drag force per unit length


(2.3) $$\begin{array}{lcr} & f=-c_{⟂}\left(u\cdot n\right)n-c_{\|}\left(u\cdot t\right)t=-c_{⟂}u+\left(c_{⟂}-c_{\|}\right)\left(u\cdot t\right)t. & \end{array}$$

The motion of the filament is then determined by balancing the elastic, active and hydrodynamic forces. For brevity, we omit the derivation of the classical EHD equations, often done through variational calculus [34], and instead directly state them:


(2.4)ψt=1c⊥(−Aψssss+msss+ψsτs+τψss)+1c‖ψs(Aψsψss−ψsms+τs),(2.5)τss−c‖c⊥ψs2τ=c⊥+c‖c⊥ψs(mss−Aψsss)+ψss(ms−Aψss).

Here τ is an elastic tension force (units of N) that enforces inextensibility. Note that the applied moment per unit length $m_{s}$ has replaced the internal moment per unit length af in Ref[34].

*Dimensionless equations*. We now apply non-dimensionalization, scaling lengths with the filament length L, time with 1/ω (a relevant angular forcing frequency) and moments and forces with A/L and $A/L^{2}$, respectively. Therefore the form of equation (2.2) for the filament velocity is unchanged,


(2.6) $$\begin{array}{lcr} & u(s,t)=\left(\begin{array}{c} -U(t)\\ V(t) \end{array}\right)+\int_{0}^{s}\left(\begin{array}{c} -\sin\;\psi (s^{\prime },t)\\ \cos\;\psi (s^{\prime },t) \end{array}\right)\psi_{t}(s^{\prime },t)ds^{\prime }, & \end{array}$$

though now 0≤s≤1 and u, U and V have (implicitly) been non-dimensionalized by scaling them with Lω. In addition, the dimensionless hydrodynamic drag force per unit length is given by


(2.7) $$\begin{array}{lcr} & f=Sp^{4}\left(-u+\frac{c_{⟂}-c_{\|}}{c_{⟂}}\left(u\cdot t\right)t\right), & \end{array}$$

with the dimensionless ‘sperm’ number Sp defined as


(2.8) $$\begin{array}{lcr} & Sp=\frac{L}{{\left(A/\omega c_{⟂}\right)}^{1/4}}=\frac{L}{l_{e}}, & \end{array}$$

where $l_{e}$ is the elastic penetration length, interpreted physically as the typical length scale over which an elastically travelling displacement wave is damped by fluid drag. Finally, we obtain the EHD equations in dimensionless form as,


(2.9)Sp4ψt=−ψssss+ψsτs+ψssτ+msss+c⊥c‖ψs(ψsψss+τs−msψs),(2.10)τss−c‖c⊥ψs2τ=c⊥+c‖c⊥ψs(−ψsss+mss)+ψss(−ψss+ms).

*Global force balance.* By noting that the total hydrodynamic force must be zero in Stokes flow, we can integrate the dimensionless hydrodynamic force density, equation (2.7), along the length of the filament, where u is given by equation (2.6) , giving a global dimensionless force balance as


(2.11) $$\begin{array}{lcr} & \begin{array}{rl} \left(\begin{array}{c} 0\\ 0 \end{array}\right)= & \int_{0}^{1}\{\left(\begin{array}{c} (1-\frac{c_{⟂}-c_{\|}}{c_{⟂}}\cos^{2}(\psi ))U+\frac{c_{⟂}-c_{\|}}{c_{⟂}}\sin(\psi )\;\cos(\psi )V\\ (-1+\frac{c_{⟂}-c_{\|}}{c_{⟂}}\sin^{2}(\psi ))V-\frac{c_{⟂}-c_{\|}}{c_{⟂}}\sin(\psi )\;\cos(\psi )U \end{array}\right)\\ & +\left(\begin{array}{c} \int_{0}^{s}\psi_{t}(s^{\prime })\;\sin(\psi (s^{\prime }))+\frac{c_{⟂}-c_{\|}}{c_{⟂}}\cos(\psi (s))\psi_{t}(s^{\prime })(\sin(\psi (s)-\psi (s^{\prime })))ds^{\prime }\\ \int_{0}^{s}-\psi_{t}(s^{\prime })\;\cos(\psi (s^{\prime }))+\frac{c_{⟂}-c_{\|}}{c_{⟂}}\sin(\psi (s))\psi_{t}(s^{\prime })(\sin(\psi (s)-\psi (s^{\prime })))ds^{\prime } \end{array}\right)\}ds, \end{array} & \end{array}$$

from which (the dimensionless) U and V will be determined.

*Leading-order asymptotics*. Next, we assume that the dimensionless forcing is small, of the form $m=\epsilon m^{\left(1\right)}$, and look to solve the problem in powers of ϵ≪1. While the following analysis is therefore rigorously valid only for small ϵ, previous results using similar analysis have demonstrated remarkable accuracy for even O(1) forcing [18]. Note that changing m to −m would have no effect on τ(s,t) or U(t), but would change the signs of ψ(s,t) and V(t). Furthermore, in the limit ϵ→0, we must have U→0 and τ→0. Therefore we deduce the following expansions in the small parameter ϵ


(2.12) $$\begin{array}{rl} \psi =\epsilon \psi^{\left(1\right)}+\epsilon^{3}\psi^{\left(3\right)}+\ldots ,\;\;\;\;\; & \;\;\;\;\;\tau =\epsilon^{2}\tau^{\left(2\right)}+\epsilon^{4}\tau^{\left(4\right)}+\ldots ,\\ U=\epsilon^{2}U^{\left(2\right)}+\epsilon^{4}U^{\left(4\right)}+\ldots ,\;\;\;\;\; & \;\;\;\;\;V=\epsilon V^{\left(1\right)}+\epsilon^{3}V^{\left(3\right)}+\ldots . \end{array}$$

Note that the $O(\epsilon^{2})$ tension τ will, classically, prove absent from the leading-order problem and can henceforth be ignored. We have also assumed that, in the limit ϵ→0, the resting straight filament is aligned with the x axis; ψ=0. Linearizing equation (2.9) gives the classical hyperdiffusion equation for linear elastohydrodynamics


(2.13) $$\begin{array}{lcr} & Sp^{4}\psi_{t}^{\left(1\right)}+\psi_{ssss}^{\left(1\right)}=m_{sss}^{\left(1\right)}, & \end{array}$$

which describes the linearized local force balance between hydrodynamic drag (first term), restoring elastic effects (second term) and active forcing (third term). In this linear limit, the dimensionless elastic restoring moment and force exerted by material at $s^{+}$ on material at $s^{-}$ are $-\psi_{s}^{\left(1\right)}$ and $-\psi_{ss}^{\left(1\right)}$, respectively.

*Boundary conditions*. In the derivation of the full EHD equations through variational calculus in [34], boundary terms demand that both the force and torque provided by the filament itself (i.e. excluding the effects of hydrodynamic drag) must be zero at both boundaries. In our dimensionless, linear system, this becomes the conditions


(2.14) $$\begin{array}{lcr} & m^{\left(1\right)}-\psi_{s}^{\left(1\right)}=m_{s}^{\left(1\right)}-\psi_{ss}^{\left(1\right)}=0. & \end{array}$$

This is because said forces and torques are provided by material at $s^{+}$ acting on material at $s^{-}$, which cannot occur at the boundaries due to the lack of further material. This provides sufficient boundary conditions for the problem, albeit dependent on the choice of m. However, we later show that these can be reduced to boundary conditions that are independent of m, by suitable integration of equation (2.13) .

*Leading order swimming speed.* Finally, considering the global force balance equation (2.11) at leading order (specifically, O(ϵ) in the y component, and $O(\epsilon^{2})$ in the x component) yields leading order expressions for V and U,


(2.15)V(1)=−∫01(∫0sψt(1)(s′)ds′)ds,(2.16)U(2)=−∫01c⊥−c‖c‖ψ(1)V(1)+(∫0sc⊥c‖ψt(1)(s′)ψ(1)(s′)+c⊥−c‖c‖ψt(1)(s′)(ψ(1)(s)−ψ(1)(s′))ds′)ds.

We also soon show that the leading-order torque balance is zero, as required for free swimming.

### (b) Integrated hyperdiffusion equation

In preparation for solving equation (2.13) for a general forcing function m using a Green’s function G, which will require setting $m^{\left(1\right)}$ to be a δ-function, we now integrate the equation three times. We define


(2.17)y(1)(s,t)=1Sp4∫0t(mss(1)(0,t′)−ψsss(1)(0,t′)) dt′+∫0sψ(1)(s′,t) ds′,(2.18)β(s,t)=∫0sy(1)(s′,t) ds′,(2.19)Ψ(s,t)=∫0sβ(s′,t) ds′.

Here, $y^{\left(1\right)}$ will soon be shown to be the leading-order vertical position of the filament as a function of s and t; β and Ψ have no discernible physical interpretation, besides their definitions in terms of $y^{\left(1\right)}$. The governing equations for these are simply $Sp^{4}y_{t}^{\left(1\right)}+y_{ssss}^{\left(1\right)}=m_{ss}^{\left(1\right)}$ for $y^{\left(1\right)}$, and $Sp^{4}\beta_{t}+\beta_{ssss}=m_{s}^{\left(1\right)}$ for β, while the equation for Ψ is


(2.20) $$\begin{array}{lcr} & Sp^{4}\Psi_{t}+\Psi_{ssss}=m^{\left(1\right)}. & \end{array}$$

We now see that the boundary conditions equation (2.14), which here are equivalent to $m^{\left(1\right)}-\Psi_{ssss}=m_{s}^{\left(1\right)}-\Psi_{sssss}=0$, reduce to


(2.21) $$\begin{array}{lcr} & \Psi_{t}=\Psi_{st}=0, & \end{array}$$

at both boundaries, and these are the boundary conditions we henceforth consider.

### (c) Calculating $V^{\left(1\right)}$ and $U^{\left(2\right)}$ and verifying global torque balance

We now obtain simple expressions for the velocity of the filament tip, for general time-periodic forcing $m^{\left(1\right)}$, by applying these governing equations and boundary conditions. Recalling equation (2.15), the vertical speed is given by


(2.22) $$\begin{array}{lcr} & \begin{array}{rl} V^{\left(1\right)} & =-\int_{0}^{1}\left(\int_{0}^{s}\psi_{t}^{\left(1\right)}(s^{\prime })ds^{\prime }\right)ds\\ & =-\frac{1}{Sp^{4}}\int_{0}^{1}\left(\int_{0}^{s}m_{s^{\prime }s^{\prime }s^{\prime }}^{\left(1\right)}(s^{\prime })-\psi_{s^{\prime }s^{\prime }s^{\prime }s^{\prime }}^{\left(1\right)}(s^{\prime })ds^{\prime }\right)ds\\ & =-\frac{1}{Sp^{4}}\int_{0}^{1}\left(m_{ss}^{\left(1\right)}(s)-m_{ss}^{\left(1\right)}(0)-\psi_{sss}^{\left(1\right)}(s)+\psi_{sss}^{\left(1\right)}(0)\right)ds\\ & =\frac{1}{Sp^{4}}\left(m_{ss}^{\left(1\right)}(0)-\psi_{sss}^{\left(1\right)}(0)\right)\;=\;y_{t}^{\left(1\right)}(0,t)\;=\;\Psi_{sst}(0,t). \end{array} & \end{array}$$

Therefore, $y^{\left(1\right)}(0,t)$ can be understood as the vertical position of the front tip at time t in this linearized limit. From the definition of $y^{\left(1\right)}$, we then see that $y^{\left(1\right)}(s,t)$ is the leading-order vertical position of the point s at time t. Finally, note that periodic forcing, i.e. periodic Ψ, will therefore yield $\left\langle V^{\left(1\right)}\right\rangle =0$, where ⟨…⟩ denotes the time-average over a period.

Similarly, we can determine the time-average of leading-order swimming speed, $U^{\left(2\right)}$. Noting that the time-average of $\psi^{\left(1\right)}\psi_{t}^{\left(1\right)}$ is zero for periodic forcing, we find


(2.23) $$\begin{array}{lcr} & \begin{array}{rl} \left\langle U^{\left(2\right)}\right\rangle & =-\frac{c_{⟂}-c_{\|}}{c_{\|}}\int_{0}^{1}\left\{\left\langle \psi^{\left(1\right)}V^{\left(1\right)}\right\rangle +\left(\int_{0}^{s}\left\langle \psi_{t}^{\left(1\right)}(s^{\prime })\psi^{\left(1\right)}(s)\right\rangle ds^{\prime }\right)\right\}ds\\ & =-\frac{c_{⟂}-c_{\|}}{c_{\|}}\int_{0}^{1}\left\langle \psi^{\left(1\right)}\left[V^{\left(1\right)}+\left(\int_{0}^{s}\psi_{t}^{\left(1\right)}(s^{\prime })ds^{\prime }\right)\right]\right\rangle ds\\ & =-\frac{c_{⟂}-c_{\|}}{c_{\|}}\int_{0}^{1}\left\langle \psi^{\left(1\right)}y_{t}^{\left(1\right)}\right\rangle ds\;=\;-\frac{c_{⟂}-c_{\|}}{c_{\|}}\int_{0}^{1}\left\langle \Psi_{sss}\Psi_{sst}\right\rangle ds. \end{array} & \end{array}$$

This is simply the classical propulsive force formula [1] divided by $c_{\|}$. Here it will be useful to define the reduced swimming speed as $U=\frac{2c_{\|}}{c_{⟂}-c_{\|}}\left\langle U^{\left(2\right)}\right\rangle$, giving


(2.24) $$\begin{array}{lcr} & U=-2\int_{0}^{1}\left\langle \Psi_{sss}\Psi_{sst}\right\rangle ds. & \end{array}$$

Finally, we can calculate the leading order global torque, measured about the tip, acting on the filament as


(2.25) $$\begin{array}{lcr} & G^{\left(1\right)}=\int_{0}^{1}y_{t}^{\left(1\right)}sds=\int_{0}^{1}\Psi_{sst}sds. & \end{array}$$

Recalling that $\Psi_{st}=\Psi_{t}=0$ at both boundaries, we integrate by parts to obtain


(2.26) $$\begin{array}{lcr} & G^{\left(1\right)}=-\int_{0}^{1}\Psi_{st}ds=0, & \end{array}$$

hence the leading-order global torque $G^{\left(1\right)}$ is indeed zero at all times.

### (d) Solving for filament motion using a Green’s function

We now consider the simple case of a moment forcing function $m^{\left(1\right)}(s,t)=R\left[f(s)e^{-i\phi (s)}e^{it}\right]$, where f is real, ϕ(s) is the (real) phase function, and the angular frequency is 1 thanks to non-dimensionalization. The corresponding solution to equation (2.20) is given by $\Psi =R\left[\Phi (s)e^{it}\right]$, where Φ is a complex function given by $Sp^{4}i\Phi +\Phi_{ssss}=f(s)e^{-i\phi (s)}$, with solution


(2.27) $$\begin{array}{lcr} & \Phi (s)=\int_{0}^{1}G(s;\xi )f(\xi )e^{-i\phi (\xi )}d\xi . & \end{array}$$

G(s;ξ) is the Green’s function of the problem, given by


(2.28) $$\begin{array}{lcr} & Sp^{4}iG+G^{\prime \prime \prime \prime }=\delta (s-\xi ), & \end{array}$$

and subject to $G=G_{s}=0$ at both boundaries. The full derivation and expression for G are given in appendix A. In particular, G is comprised entirely of the four natural modes $e^{ks}$, where $k^{4}=-Sp^{4}i$. Therefore, the solution $\Psi =R\left[\Phi (s)e^{it}\right]$ for general forcing function $m^{\left(1\right)}(s,t)=R\left[f(s)e^{-i\phi (s)}e^{it}\right]$ is written as


(2.29) $$\Psi (s,t)=R\left[e^{it}\int_{0}^{1}G(s;\xi )f(\xi )e^{-i\phi (\xi )}\;d\xi \right].$$

Recalling that $y^{\left(1\right)}=\Psi_{ss}$ and $\psi^{\left(1\right)}=\Psi_{sss}$, this then allows us to express the filament shape in terms of the derivatives of G with respect to s,


(2.30) $$y^{\left(1\right)}(s,t)=R\left[e^{it}\int_{0}^{1}G^{\prime \prime }(s;\xi )f(\xi )e^{-i\phi (\xi )}\;d\xi \right],$$

or equivalently,


(2.31) $$\psi^{\left(1\right)}(s,t)=R\left[e^{it}\int_{0}^{1}G^{\prime \prime \prime }(s;\xi )f(\xi )e^{-i\phi (\xi )}\;d\xi \right].$$

Note that G, and therefore Ψ, $y^{\left(1\right)}$ and $\psi^{\left(1\right)}$, are dependent upon the dimensionless sperm number Sp that parameterizes the problem. We omit the explicit dependence on Sp for brevity, but proceed with the understanding that any calculation is done for a specific value of Sp. In particular, any optima that we identify are the optima for a specific value of Sp, and variation of Sp will be necessary to determine global optima.

### (e) Calculating the swimming speed using G

The result in equation (2.24) can be used to calculate the swimming speed when the filament shape is known. We would first need to calculate Ψ and its derivatives, perform a time-average, and finally an integral in s. However, it is possible to circumvent calculating the filament shape, and instead calculate the swimming speed directly from the forcing function and G, using a double integral. For general moment forcing $m^{\left(1\right)}(s,t)=R\left[f(s)e^{-i\phi (s)}e^{it}\right]$, we show in appendix B that U is given by


(2.32) $$\begin{array}{lcr} & U=-\int_{\xi_{1}=0}^{1}\int_{\xi_{2}=0}^{1}f(\xi_{1})ℑ\left[G^{\prime }(\xi_{1};\xi_{2})e^{i(\phi (\xi_{1})-\phi (\xi_{2}))}\right]f(\xi_{2})d\xi_{2}d\xi_{1}. & \end{array}$$

Importantly, the derivation of this equation in appendix B involves a time-average over a period. Therefore, if higher frequency modes are present within $m^{\left(1\right)}$ (i.e. ω=2,3,4,… under the current non-dimensionalization, which is applied with regard to the fundamental mode) then their interactions will be averaged and vanish since, for example ⟨sin⁡(at)sin⁡(bt)⟩=0 when a and b are distinct integers. Similarly, any constant forcing (i.e. ω=0) will have no effect on the swimming speed, since constant forcing cannot produce swimming, nor can any interactions between this constant forcing and non-zero frequency modes. Noting that each mode will have its own non-dimensionalization, and therefore require its own unique re-dimensionalization, we deduce that the overall dimensional swimming speed can be obtained simply as the sum of the dimensional swimming speeds corresponding to each individual temporal mode of $m^{\left(1\right)}$. Obviously, this equation for the swimming speed is not very intuitive due to the complex values of G and $e^{i\phi }$. We can further define real symmetric and antisymmetric functions as


(2.33)Gs(ξ1,ξ2)=−12(ℑ[G′(ξ1;ξ2)]+ℑ[G′(ξ2;ξ1)]),(2.34)Ga(ξ1,ξ2)=−12(R[G′(ξ1;ξ2)]−R[G′(ξ2;ξ1)]),

and these enable us to rewrite equation (2.32) as


(2.35) $$\begin{array}{lcr} & U=\int_{\xi_{1}=0}^{1}\int_{\xi_{2}=0}^{1}f(\xi_{1})G_{swim}(\xi_{1},\xi_{2})f(\xi_{2})d\xi_{2}d\xi_{1}, & \end{array}$$

where


(2.36) $$G_{swim}(\xi_{1},\xi_{2})=G_{s}(\xi_{1},\xi_{2})\cos\left(\phi (\xi_{1})-\phi (\xi_{2})\right)+G_{a}(\xi_{1},\xi_{2})\sin\left(\phi (\xi_{1})-\phi (\xi_{2})\right)$$

is the (real) swimming speed function. The result in equation (2.35) is the first main result of this paper, showing the direct link between forcing (function f) and swimming (reduced speed U).

Once again, note that $G_{swim}$ is dependent upon Sp, and also on the phase function ϕ, though we omit these dependences from the notation for brevity. By construction, $G_{swim}$ is real and symmetric for any phase function ϕ. Once $G_{s}$ and $G_{a}$ are calculated, U can easily be numerically evaluated for any forcing magnitude f(s) and phase ϕ(s).

## 3. Modal analysis

By deriving and solving the classical forced hyperdiffusion equation for filament motion, we have determined equation (2.35) for the swimming speed U of the filament entirely in terms of the forcing function f and the real symmetric function $G_{swim}$ that depends on the sperm number Sp and phase function ϕ.

There are, however, two practical concerns regarding equation (2.35). First, this equation has a quadratic computational complexity. If $G_{swim}$ has been computed, and f is known, and the integrals are evaluated using some N-point numerical integration scheme (e.g. trapezium approximation), then evaluating U will usually be an $O(N^{2})$ process. Second, if we are interested in maximizing U over choice of f, there is no clear way to do this using equation (2.35).

We now show that both concerns can be addressed by exploiting the real symmetric nature of $G_{swim}$ and deriving a modal analysis of the system. We show that U can be well approximated using an O(N) or even O(1) process, and optimization techniques for the choice of f become available.

### (a) Eigenfunctions and eigenvalues: theory

The key point that allows us to derive a modal analysis of this problem is to note that, as the continuum extension of a real symmetric matrix, $G_{swim}$ has an infinite basis of orthonormal eigenfunctions $g_{n}(\xi )$ and corresponding eigenvalues $\lambda_{n}$, dependent upon Sp and ϕ, and given by


(3.1) $$\begin{array}{lcr} & \int_{0}^{1}G_{swim}(\xi_{1},\xi_{2})g_{n}(\xi_{2})\;d\xi_{2}=\lambda_{n}g_{n}(\xi_{1}),\;\int_{0}^{1}g_{m}(\xi )g_{n}(\xi )\;d\xi =\delta_{mn}. & \end{array}$$

We can express f in terms of this basis, and hence express the swimming speed from equation (2.35) via this modal approach, leading to


(3.2) $$\begin{array}{lcr} & f(\xi )=\sum_{n=1}^{\infty }a_{n}g_{n}(\xi ),\;a_{n}=\int_{0}^{1}f(\xi )g_{n}(\xi )\;d\xi ,\;U=\sum_{n=1}^{\infty }a_{n}^{2}\lambda_{n}. & \end{array}$$

Since eigenvalues typically decay in magnitude as n becomes large, a finite truncation of these series is usually sufficient to produce accurate results.

### (b) Eigenfunctions and eigenvalues: computation

**Figure 2.**
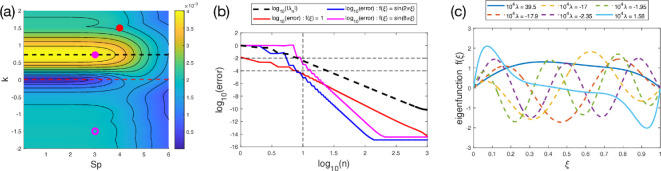
Modal approach under travelling wave forcing. (a) Heatmap of the largest positive eigenvalue for various values of Sp and k, with k=0 (dashed red line) and the optimal k≈0.72 (dashed black line) indicated. Examples of spermatozoa swimming through *in vitro* fertilization medium (red, k=1.5, Sp=4 [4,36,37]) and a near-optimal active filament (solid pink, k=0.72, Sp=3) are also shown, as well as optimal swimming in the same direction as wave propagation (hollow pink, k=−1.5, Sp=3). (b) Log–log plot (base 10) showing the errors incurred as the truncation of equation (3.2) is varied, for a near-optimal filament (k=0.72, Sp=3). Eigenvalues are arranged in order of decreasing magnitude, normalized relative to the largest/first eigenvalue, and their decay as n increases is given (thick dashed black line). Also plotted is the relative error incurred by truncating equation (3.2) at the *n*-th term, normalized relative to the exact swimming speed calculated using equation (2.35), for simple forcing functions f(ξ)≡1 (red solid line), f(ξ)=sin⁡(2πξ) (blue) and f(ξ)=sin⁡(8πξ) (pink). (c) The eigenfunctions for the six eigenvalues with largest magnitudes, for the same near-optimal filament, normalized using the fixed forcing magnitude constraint, equation (3.5). The first and sixth eigenvalues are positive (solid lines) while the others are negative (dashed lines).

Algebraically calculating the eigenfunctions and eigenvalues of $G_{swim}$ is often technically possible by exploiting the definition of Green’s function as the solution to a differential equation (see Example B below). However, it is usually easier to calculate them numerically by discretizing $G_{swim}$ into an N×N real symmetric matrix and the eigenfunction $g_{k}$ into a real vector of length N,


(3.3) $$\begin{array}{lcr} & G_{mn}^{matrix}=G_{swim}\left(\frac{2m-1}{2N},\frac{2n-1}{2N}\right),\;g_{k,n}^{vector}=g_{k}\left(\frac{2n-1}{2N}\right), & \end{array}$$

where 1≤m,n≤N. The discretized eigenfunctions (eigenvectors in this context) and corresponding eigenvalues of equation (3.1) are therefore given by approximating the integral numerically as


(3.4) $$\begin{array}{lcr} & G^{matrix}g_{k}^{vector}\approx N\lambda_{k}g_{k}^{vector}, & \end{array}$$

and so the eigenfunctions and eigenvalues can easily be obtained via standard methods of computing the eigenvectors and eigenvalues of a real symmetric matrix. Note that N must be large enough to accurately capture the behaviour of $G_{swim}$ and its eigenfunctions; in practice we find that setting N=100 comfortably achieves this, in the sense that increasing N further, even to N=1000, had no discernible effect on any of the results or figures discussed below. Setting N=100 enables $G^{matrix}$ to be evaluated, and its eigenfunctions and eigenvalues calculated, practically instantly on a laptop computer. We find that the eigenvalues typically decay quite quickly, and only a handful of eigenfunctions need be considered in practice. Importantly, the number of relevant eigenvalues does not change noticeably when increasing N, see figure 2*b* below. Of course, the eigenfunctions and eigenvalues depend on the phase function ϕ and the sperm number Sp. While Sp is something that can be continuously varied to find an optimum, the function ϕ is often set by physical or biological context, as we will soon demonstrate with two key examples.

### (c) Using calculus of variations to prove the eigenfunctions produce optimal swimming

To continue with this modal analysis, we first seek an intuitive understanding of the eigenfunctions and eigenvalues obtained above, in particular how they provide optimal choices for the forcing function. We first note that f must be constrained in some way, otherwise we could simply, for example, double f to quadruple U. Various physical and biological constraints may be relevant, such as having a fixed rate of doing work (particularly relevant for biological cells such as spermatozoa) or engineering constraints that limit the choice of f (relevant for artificial swimmers).

From a mathematical perspective, the simplest constraint is one of fixed average magnitude,


(3.5) $$\begin{array}{lcr} & \int_{0}^{1}f(\xi {)}^{2}\;d\xi =1, & \end{array}$$

which can be interpreted physically as the filament having a fixed total (squared) moment forcing. We then consider the variational optimization of the swimming speed subject to this fixed forcing magnitude, yielding the Lagrangian


(3.6) $$\begin{array}{lcr} & L=\int_{\xi_{1}=0}^{1}\int_{\xi_{2}=0}^{1}f(\xi_{1})G_{swim}(\xi_{1},\xi_{2})f(\xi_{2})\;d\xi_{2}d\xi_{1}-\lambda \left[\int_{0}^{1}f(\xi {)}^{2}\;d\xi -1\right], & \end{array}$$

where λ acts as the Lagrange multiplier of the variational problem, enforcing the constraint in equation (3.5) (i.e. the bracketed term is zero). Applying the standard perturbation f↦f+δf yields a linear change in the Lagrangian given by


(3.7) $$\begin{array}{lcr} & \frac{1}{2}\delta L=\int_{\xi_{1}=0}^{1}\int_{\xi_{2}=0}^{1}\delta f(\xi_{1})G_{swim}(\xi_{1},\xi_{2})f(\xi_{2})\;d\xi_{2}d\xi_{1}-\lambda \int_{0}^{1}\delta f(\xi )f(\xi )\;d\xi . & \end{array}$$

By writing this as


(3.8) $$\begin{array}{lcr} & \frac{1}{2}\delta L=\int_{\xi_{1}=0}^{1}\delta f(\xi_{1})\left[\int_{\xi_{2}=0}^{1}G_{swim}(\xi_{1},\xi_{2})f(\xi_{2})\;d\xi_{2}-\lambda f(\xi_{1})\right]\;d\xi_{1}, & \end{array}$$

we establish the solution to this variational problem as the function f (obeying the fixed forcing magnitude constraint) satisfying


(3.9) $$\begin{array}{lcr} & \int_{\xi_{2}=0}^{1}G_{swim}(\xi_{1},\xi_{2})f(\xi_{2})\;d\xi_{2}=\lambda f(\xi_{1}). & \end{array}$$

The result in equation (3.9) shows that the eigenfunctions f of $G_{swim}$ are precisely the choice of forcing function which provide (local) optima to this variational problem. In other words, setting f to be an eigenfunction of $G_{swim}$ necessarily yields a local maximum (or minimum) for the swimming speed. The global maximum (for each particular value of Sp and ϕ) can then be identified as the eigenfunction with the largest (positive) eigenvalue, which can then be maximized over all acceptable choices of Sp and ϕ to obtain the true maximum swimming speed. Alternatively, swimming in the opposite direction can be maximized by choosing the eigenfunction with the largest (in magnitude) negative eigenvalue. It should be noted that this simple choice of f is only optimal when the constraint upon f truly is a fixed magnitude constraint; a variety of other constraints may be relevant, such as a fixed rate of doing work, or constraints upon f itself (see Example B below). In such cases, the full suite of eigenfunctions will have to be considered in general, though typically all but a handful of these can be neglected by virtue of small eigenvalues.

### (d) Example A: travelling wave forcing

We now consider the application of our modal approach to two relevant examples. A biologically relevant situation is that of a travelling wave of forcing [4]. If we set ϕ(s)=2πks for some constant k, the overall moment forcing is then given by


(3.10) $$m^{\left(1\right)}(s,t)=R\left[f(s)e^{i(t-2\pi ks)}\right].$$

This corresponds to a forcing wave of wavelength 1/k (i.e. there are k wavelengths on the flagellum) travelling at speed 1/2πk in the backwards direction, i.e. from the proximal end (s=0) to the distal end (s=1). This is approximately observed in real spermatozoa, both in high-viscosity, viscoelastic cervical mucus substitute (k≈2−3, Sp≈24) and low-viscosity *in vitro* fertilization medium with viscosity similar to that of water (k≈1.5, Sp≈4) [4,36,37].

With the moment forcing in equation (3.10), the swimming speed function $G_{swim}$ is given by


(3.11) $$\begin{array}{lcr} & G_{swim}(\xi_{1},\xi_{2})=G_{s}(\xi_{1},\xi_{2})\;\cos\left(2\pi k\left(\xi_{1}-\xi_{2}\right)\right)+G_{a}(\xi_{1},\xi_{2})\;\sin\left(2\pi k\left(\xi_{1}-\xi_{2}\right)\right). & \end{array}$$

Note that the forcing strength f is stationary and does not move with the wave. One naturally expects swimming to occur in the direction opposite to the direction of wave propogation, i.e. Uk>0 under the current sign convention, and this is observed in spermatozoa [4,36,37], with backwards travelling waves (k>0) generating forwards swimming (U>0). In addition, k<0 corresponds to forwards travelling waves, which are generally not biologically observed.

#### (i) Results of modal analysis

*Optimal swimming mode*. For each Sp and k, we next apply the above modal approach to compute the eigenvalues of equation (3.11). The magnitude of largest positive eigenvalue is plotted in figure 2*a* as a function of Sp and k (allowing both signs for k). For each value of the parameters, this corresponds to the optimal swimming speed under the constraint of fixed forcing magnitude. Note that, given the distributed forcing, swimming speed does not tend to zero as Sp→0, in contrast to classical results for single-point actuation [17]. However, the swimming speed does tend to zero as Sp becomes large, corresponding to elastic waves that decay over very short dimensionless length scales.

We observe that, in accordance with the intuition that Uk>0 in the biological world (i.e. swimming occurs in the direction opposite to wave propagation), the optimal k (i.e. that which maximizes forward swimming) is positive, approximately k=0.72 (dashed black line in figure 2*a*). In fact, there is a large region of near-optimality, for a range of values of both k and Sp, and the dependence on Sp within this region is extremely weak. This means that there is a great deal of freedom in choosing k and especially Sp to optimize the forward swimming speed. Note that the actual optimum has Sp→0, which is obviously physically impossible. Therefore we often select a ‘near-optimum’ (solid pink dot in figure 2*a*) that could correspond to a real swimmer, without any notable sacrifice to swimming speed.

Remarkably, the conditions for near-optimal swimming of this isolated filament (solid pink dot in figure 2*a*) are similar to the conditions observed in real spermatozoa swimming through water-like *in vitro* fertilization medium (red dot in figure 2*a*) [4,36,37]. This suggests that despite the various assumptions made, such as the linearization and lack of a head, an active filament model reasonably approximates biological spermatozoa.

Interestingly, Uk<0 is also possible, and surprisingly effective (local optimum in the region Uk<0 shown by the hollow pink dot in figure 2*a*), with forwards travelling waves able to generate approximately half the maximum speed compared with the Uk>0 case.

*Superposition of modes*. If f cannot be freely chosen, or is not subject to a fixed forcing magnitude, we must use the full suite of eigenfunctions and eigenvalues. Fortunately, the eigenvalues decay rapidly, as shown in figure 2*b* (thick dashed black line) for a near-optimal filament with k=0.72 and Sp=3. Of course, a small eigenvalue can still contribute significantly to U if the corresponding coefficient $a_{n}$ is large enough. For a typical forcing function f(ξ)≡1, approximately observed in spermatozoa [4], figure 2*b* also shows that the relative errors in approximating U using a truncation of equation (3.2), compared with using the exact expression in equation (2.35), also decays rapidly, and only a few terms need be retained to obtain an accurate result.

For comparison, we also show in figure 2*b* the corresponding results obtained by setting f(ξ)=sin⁡(2πξ) and f(ξ)=sin⁡(8πξ). Overall, only a small truncation is needed, with just 10 terms being sufficient to obtain an error of less than 0.01% for both f(ξ)≡1 and f(ξ)=sin⁡(2πξ), with the higher-frequency forcing f(ξ)=sin⁡(8πξ) incurring an error of approximately 0.1% using the same number of modes.

This increase in error induced by higher frequency forcing can be understood physically by examining the form of the eigenfunctions. The six eigenvalues with largest magnitudes, and their corresponding eigenfunctions (normalized using the fixed forcing magnitude constraint, equation (3.5)), are shown in figure 2*c*, again for a near-optimal filament with k=0.72 and Sp=3. Despite the fact that the forcing wave is travelling backwards (i.e. k>0) four of these six eigenvalues are negative (the largest eigenvalue is positive, however, and more than twice the magnitude of any other eigenvalue). Furthermore, the negative eigenvalues appear to have eigenfunctions which are significantly more oscillatory than their counterparts for positive eigenvalues, and the eigenfunction of the largest eigenvalue is the least oscillatory of all. In fact, the frequency of the eigenmodes increases as the magnitudes of the eigenvalues decrease, explaining the greater errors observed for higher frequency forcing seen in figure 2*b*. Finally, note that the eigenfunction of the largest eigenvalue is close to the actual forcing observed in real spermatozoa [4].

It is important to note that these results do not discernibly change when increasing N, hence the number of relevant eigenfunctions and eigenvalues does not change as N is further increased. We can therefore generally truncate equation (3.2) to a fairly small number of modes, while still retaining excellent accuracy in calculating the swimming speed.

#### (ii) Computational considerations

When f can be freely varied, and is subject to a fixed forcing magnitude, we immediately obtain the optimal f for fixed Sp and k as the eigenfunction with the largest eigenvalue. Otherwise, we use a finite truncation of equation (3.2), and this provides multiple computational advantages compared with standard methods of calculating U.

For any given f, the swimming speed could be evaluated without the use of the modal approach, using equation (2.35), by performing a double integral. Assuming said integrals are calculated numerically, using some N-point scheme (such as a trapezium approximation), this represents a computational complexity of $O(N^{2})$. However, using the modal approach, with a finite truncation of the eigenfunctions in equation (3.2) (e.g. retaining the 10 largest-in-magnitude eigenvalues), we instead only need to calculate a fixed number of single integrals, and so the overall computational complexity of calculating U is O(N), with a slight trade-off of accuracy in the result due to the neglected eigenfunctions. Of course, this requires us to know the eigenfunctions and eigenvalues; however, once these are calculated, they can be applied to calculate U in O(N) time for any f.

This advantage is even more striking if, instead of choosing f and then calculating integrals to identify the coefficients $a_{n}$ of equation (3.2), we do the converse: select the $a_{n}$, and use these to calculate f. Not only does this fully circumvent the need to calculate the integrals, enabling U to be computed with O(1) complexity, but it reduces the phase space of f from an infinite-dimensional one (where f can be continuously varied) to one of finite dimensions. Then the optimal f, for that particular Sp and k, can be identified easily by maximizing U through variation of the $a_{n}$.

Clearly, the actual procedure to calculate U will depend on the constraints imposed on f. Furthermore, these methods do not provide any way to maximize U over all Sp and k.

### (e) Example B: monophasic forcing

Having considered an example that is biologically relevant, we now turn to one that is applicable to the design of artificial swimmers. Due to the difficulties of engineering on such small scales, forcing is typically simple in form. Two notable examples of experimental artificial swimmers are the filament made of magnetic beads used in [25], and the polymeric flagellum actuated by cardiomyocytes used in [30]. In both of these cases, forcing generally acts in phase, ϕ≡0 (equivalent to the k=0 case in the previous example), with the former applying distributed forcing and the latter utilizing point actuation. When taking ϕ≡0, $G_{swim}$ is given simply by


(3.12) $$\begin{array}{lcr} & G_{swim}(\xi_{1},\xi_{2})=G_{s}(\xi_{1},\xi_{2})=-\frac{1}{2}\left(ℑ\left[G^{\prime }(\xi_{1};\xi_{2})\right]+ℑ\left[G^{\prime }(\xi_{2};\xi_{1})\right]\right). & \end{array}$$

In this section, we apply our modal approach to develop optimization techniques for such artificial swimmers. We find, remarkably, that only (exactly) four of the eigenvalues are non-zero. Two of these are simply negatives of the other two, their eigenfunctions being reflections, due to the symmetry of the filament. We start below by analytically proving that only four of the eigenvalues are non-zero, followed by developing an analytic method to calculate the corresponding eigenfunctions. We then apply these results to establish exact formulae for the swimming speed U, before making various approximations to optimize the configuration of an artificial swimmer, and demonstrating this using simple examples.

#### (i) Only four eigenvalues are non-zero

Given the swimming speed function in equation (3.12), the eigenfunction f with eigenvalue λ satisfies


(3.13) $$\begin{array}{rl} \lambda f(\xi_{1}) & =\int_{0}^{1}G_{swim}(\xi_{1},\xi_{2})f(\xi_{2})\;d\xi_{2}\\ & =\frac{1}{2}ℑ\left[\int_{0}^{1}-G^{\prime }(\xi_{1};\xi_{2})f(\xi_{2})-G^{\prime }(\xi_{2};\xi_{1})f(\xi_{2})\;d\xi_{2}\right]\\ & =\frac{1}{2}ℑ\left[-\frac{\partial }{\partial \xi_{1}}\left(\int_{0}^{1}G(\xi_{1};\xi_{2})f(\xi_{2})\;d\xi_{2}\right)+\int_{0}^{1}G(\xi_{2};\xi_{1})\frac{\partial }{\partial \xi_{2}}\left(f(\xi_{2})\right)\;d\xi_{2}\right]\\ & =\frac{1}{2}ℑ\left[-I_{1}^{\prime }(\xi_{1})+I_{2}(\xi_{1})\right]\\ & =R\left[I_{3}\right], \end{array}$$

where $I_{1}$ and $I_{2}$ are the solutions to the differential equations


(3.14)Sp4iI1(ξ1)+I1⁗(ξ1)=f(ξ1),(3.15)Sp4iI2(ξ1)+I2⁗(ξ1)=f′(ξ1),

and $I_{3}=\frac{1}{2}i\left(I_{1}^{\prime }-I_{2}\right)$ satisfies the equation


(3.16) $$\begin{array}{lcr} & Sp^{4}iI_{3}(\xi_{1})+I_{3}^{\prime \prime \prime \prime }(\xi_{1})=0, & \end{array}$$

with $I(0)=I(1)=I^{\prime }(0)=I^{\prime }(1)=0$ for both $I_{1}$ and $I_{2}$.

Defining, as in appendix A, $\eta =e^{-\pi i/8}$, we therefore deduce that


(3.17) $$I_{3}(\xi_{1})=Ae^{Sp\eta \xi_{1}}+Be^{Sp\eta i\xi_{1}}+Ce^{-Sp\eta \xi_{1}}+De^{-Sp\eta i\xi_{1}}$$

consists entirely of the natural modes of the filament. Therefore, since the eigenfunction f must satisfy $\lambda f(\xi_{1})=R\left[I_{3}\right]$, we deduce that λ=0 whenever f contains a non-natural mode, and so there are only finitely many eigenfunctions with non-zero eigenvalues, each constructed entirely using the natural modes. Using complex coefficients, $I_{3}$ has only four modes, while using real coefficients, f has eight modes. Note, however, that $I_{3}(0)=I_{3}(1)=0$, which reduces the number of independent modes of $I_{3}$ to two, therefore reducing the number of independent modes of f to four. Hence there are at most four eigenfunctions f with non-zero eigenvalue, and by symmetry, two of these eigenfunctions will simply be reflections of the other two, their eigenvalues being negatives.

We can identify the $I_{3}$ corresponding to each mode of f, and equating modes then results in an eigenvector problem, allowing us to find the eigenfunctions f and their corresponding eigenvalues. This can be done using a standard method (see appendix C) to obtain an 8×8 system with four zero eigenvalues. Alternatively, as we now show, we can instead incorporate the boundary conditions for $I_{3}$ directly into the eigenfunction calculation to obtain a simpler 4×4 system that fully identifies the eigenfunctions and eigenvalues, and demonstrates their symmetry.

#### (ii) Analytic calculation of eigenvalues

The function $I_{3}$ must obey $I_{3}(0)=I_{3}(1)=0$, and therefore, by expressing two of the coefficients in terms of the other two, we find that, regardless of f, we can express $I_{3}$ in the form


(3.18) $$I_{3}(\xi_{1})=Af_{s}(\xi_{1})+Bf_{a}(\xi_{1})$$

for symmetric ($f_{s}(1-\xi_{1})=f_{s}(\xi_{1})$) and antisymmetric ($f_{a}(1-\xi_{1})=-f_{a}(\xi_{1})$) functions


(3.19)fs(ξ1)=eSpηξ1+eSpηe−Spηξ11+eSpη−eSpηiξ1+eSpηie−Spηiξ11+eSpηi,(3.20)fa(ξ1)=eSpηξ1−eSpηe−Spηξ11−eSpη−eSpηiξ1−eSpηie−Spηiξ11−eSpηi.

The forcing f must also have this form to be an eigenfunction with non-zero eigenvalue, and in particular we can calculate $I_{1}$ and $I_{2}$ for the four different modes of f. For $f(\xi )=R\left[bf_{s}(\xi_{1})\right]$, where b=1 or b=i, we have


(3.21) $$\begin{array}{lcr} & \begin{array}{rl} I_{1}^{\left(b,s\right)}(\xi_{1}) & =\frac{b\xi_{1}}{8Sp^{3}\eta^{3}}\left(\frac{e^{Sp\eta \xi_{1}}-e^{Sp\eta }e^{-Sp\eta \xi_{1}}}{1+e^{Sp\eta }}-i\frac{e^{Sp\eta i\xi_{1}}-e^{Sp\eta i}e^{-Sp\eta i\xi_{1}}}{1+e^{Sp\eta i}}\right)+\frac{b^{*}f_{s}(\xi_{1}{)}^{*}}{4Sp^{4}i}\\ & +A_{1}^{\left(b,s\right)}e^{Sp\eta \xi_{1}}+B_{1}^{\left(b,s\right)}e^{Sp\eta i\xi_{1}}+C_{1}^{\left(b,s\right)}e^{-Sp\eta \xi_{1}}+D_{1}^{\left(b,s\right)}e^{-Sp\eta i\xi_{1}},\\ I_{2}^{\left(b,s\right)}(\xi_{1}) & =\frac{b\xi_{1}}{8Sp^{2}\eta^{2}}\left(\frac{e^{Sp\eta \xi_{1}}+e^{Sp\eta }e^{-Sp\eta \xi_{1}}}{1+e^{Sp\eta }}+\frac{e^{Sp\eta i\xi_{1}}+e^{Sp\eta i}e^{-Sp\eta i\xi_{1}}}{1+e^{Sp\eta i}}\right)+\frac{b^{*}f_{s}^{\prime }(\xi_{1}{)}^{*}}{4Sp^{4}i}\\ & +A_{2}^{\left(b,s\right)}e^{Sp\eta \xi_{1}}+B_{2}^{\left(b,s\right)}e^{Sp\eta i\xi_{1}}+C_{2}^{\left(b,s\right)}e^{-Sp\eta \xi_{1}}+D_{2}^{\left(b,s\right)}e^{-Sp\eta i\xi_{1}}. \end{array} & \end{array}$$

In addition, if $f(\xi )=R\left[bf_{a}(\xi_{1})\right]$, where b=1 or b=i, we obtain


(3.22) $$\begin{array}{rl} I_{1}^{\left(b,a\right)}(\xi_{1}) & =\frac{b\xi_{1}}{8Sp^{3}\eta^{3}}\left(\frac{e^{Sp\eta \xi_{1}}+e^{Sp\eta }e^{-Sp\eta \xi_{1}}}{1-e^{Sp\eta }}-i\frac{e^{Sp\eta i\xi_{1}}+e^{Sp\eta i}e^{-Sp\eta i\xi_{1}}}{1-e^{Sp\eta i}}\right)+\frac{b^{*}f_{a}(\xi_{1}{)}^{*}}{4Sp^{4}i}\\ & +A_{1}^{\left(b,a\right)}e^{Sp\eta \xi_{1}}+B_{1}^{\left(b,a\right)}e^{Sp\eta i\xi_{1}}+C_{1}^{\left(b,a\right)}e^{-Sp\eta \xi_{1}}+D_{1}^{\left(b,a\right)}e^{-Sp\eta i\xi_{1}},\\ I_{2}^{\left(b,a\right)}(\xi_{1}) & =\frac{b\xi_{1}}{8Sp^{2}\eta^{2}}\left(\frac{e^{Sp\eta \xi_{1}}-e^{Sp\eta }e^{-Sp\eta \xi_{1}}}{1-e^{Sp\eta }}+\frac{e^{Sp\eta i\xi_{1}}-e^{Sp\eta i}e^{-Sp\eta i\xi_{1}}}{1-e^{Sp\eta i}}\right)+\frac{b^{*}f_{a}^{\prime }(\xi_{1}{)}^{*}}{4Sp^{4}i}\\ & +A_{2}^{\left(b,a\right)}e^{Sp\eta \xi_{1}}+B_{2}^{\left(b,a\right)}e^{Sp\eta i\xi_{1}}+C_{2}^{\left(b,a\right)}e^{-Sp\eta \xi_{1}}+D_{2}^{\left(b,a\right)}e^{-Sp\eta i\xi_{1}}. \end{array}$$

Here there are 32 coefficients that must be evaluated by applying the boundary conditions on $I_{1}$ and $I_{2}$. According to equations (3.14) and (3.15), and the definition of $I_{3}$, we see that a symmetric f results in an antisymmetric $I_{3}$, and an antisymmetric f results in a symmetric $I_{3}$. Recalling the form that $I_{3}$ must take, equation (3.18), and that the non-natural modes of $I_{1}$ and $I_{2}$ vanish in $I_{3}$, we deduce that


(3.23)I3(b,s)(ξ1)=12i((I1(b,s))′−I2(b,s))=E3(b,s)fa(ξ1),(3.24)I3(b,a)(ξ1)=12i((I1(b,a))′−I2(b,a))=E3(b,a)fs(ξ1),

where each of the new coefficients is given by


(3.25)E3(b,a)=12i(1+eSpη)(SpηA1(b,a)−A2(b,a)),(3.26)E3(b,s)=12i(1−eSpη)(SpηA1(b,s)−A2(b,s)).

These four coefficients can be calculated for any given Sp, and this gives us a fully determined system. If we write the eigenfunction as


(3.27) $$f(\xi_{1})=A_{f}R\left[f_{s}(\xi_{1})\right]+B_{f}R\left[f_{a}(\xi_{1})\right]+C_{f}R\left[if_{s}(\xi_{1})\right]+D_{f}R\left[if_{a}(\xi_{1})\right],$$

then this results in an $I_{3}$ given by


(3.28) $$\begin{array}{lcr} & I_{3}(\xi_{1})=\left(A_{f}E_{3}^{\left(1,s\right)}+C_{f}E_{3}^{\left(i,s\right)}\right)f_{a}(\xi_{1})+\left(B_{f}E_{3}^{\left(1,a\right)}+D_{f}E_{3}^{\left(i,a\right)}\right)f_{s}(\xi_{1}). & \end{array}$$

Recalling that the coefficients of f are real, and that $\lambda f=R\left[I_{3}\right]$, we finally obtain an eigenvector problem


(3.29) $$\begin{array}{r} \lambda \left[\begin{array}{c} A_{f}\\ B_{f}\\ C_{f}\\ D_{f} \end{array}\right]=\left[\begin{array}{cccc} 0 & R\left[E_{3}^{\left(1,a\right)}\right] & 0 & R\left[E_{3}^{\left(i,a\right)}\right]\\ R\left[E_{3}^{\left(1,s\right)}\right] & 0 & R\left[E_{3}^{\left(i,s\right)}\right] & 0\\ 0 & I\left[E_{3}^{\left(1,a\right)}\right] & 0 & I\left[E_{3}^{\left(i,a\right)}\right]\\ I\left[E_{3}^{\left(1,s\right)}\right] & 0 & I\left[E_{3}^{\left(i,s\right)}\right] & 0 \end{array}\right]\left[\begin{array}{c} A_{f}\\ B_{f}\\ C_{f}\\ D_{f} \end{array}\right]. \end{array}$$

This equation can easily be solved for the coefficients of f, therefore identifying the eigenfunctions and eigenvalues. Furthermore, we see that any solution has the property that a change $B_{f}\mapsto -B_{f},D_{f}\mapsto -D_{f},\lambda \mapsto -\lambda$ still satisfies this equation, which is equivalent to a change f(ξ)↦f(1−ξ) with negative eigenvalue, as required by symmetry of the filament.

#### (iii) Results of modal analysis

Having shown that only four eigenvalues are non-zero, and derived a method by which to calculate them analytically, we now present these results, and exploit them to calculate the swimming speed. Denoting the two positive eigenvalues by $\lambda_{+}$ and $\lambda_{-}$, with corresponding eigenfunctions $g_{+}$ and $g_{-}$, respectively, equation (3.2) simplifies to


(3.30) $$\begin{array}{rl} U=\left[{\left(\int_{0}^{1}f(s)g_{+}(s)\;ds\right)}^{2}-{\left(\int_{0}^{1}f(s)g_{+}(1-s)\;ds\right)}^{2}\right]\lambda_{+} & \\ +\left[{\left(\int_{0}^{1}f(s)g_{-}(s)\;ds\right)}^{2}-{\left(\int_{0}^{1}f(s)g_{-}(1-s)\;ds\right)}^{2}\right]\lambda_{-} & . \end{array}$$

In particular, if the artificial filament is powered by M discrete actuators, modelled as δ-functions with strengths $F_{m}$ and positions $\xi_{m}$, this formula can be written as


(3.31) $$\begin{array}{rl} U=\left[{\left(\sum_{m=1}^{M}F_{k}g_{+}(\xi_{k})\right)}^{2}-{\left(\sum_{m=1}^{M}F_{k}g_{+}(1-\xi_{k})\right)}^{2}\right]\lambda_{+} & \\ +\left[{\left(\sum_{m=1}^{M}F_{k}g_{-}(\xi_{k})\right)}^{2}-{\left(\sum_{m=1}^{M}F_{k}g_{-}(1-\xi_{k})\right)}^{2}\right]\lambda_{-} & . \end{array}$$

These equations yield significant results, enabling U to be determined through only four sums or integrals. Again, this represents a reduction in complexity from $O(N^{2})$ to O(N) (N discretization points for a continuous f) or from $O(M^{2})$ to O(M) (number M of discrete actuators) compared with equation (2.35). However, while useful for computation of U for a particular f and Sp, these equations are not, *a priori*, conducive to analysis, nor to any optimization over f and Sp besides brute-force methods, and thus require further simplification.

#### (iv) Finding the optimal sperm number and neglecting the smaller eigenvalue

**Figure 3.**
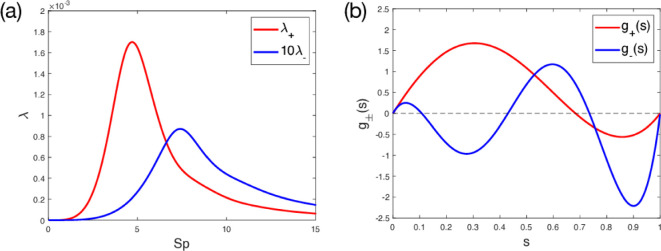
Eigenvalues and eigenfunctions for monophasic forcing. (a) The large (red) and small (blue) eigenvalues, $\lambda_{+}$ and $\lambda_{-}$, respectively, for monophasic forcing, ϕ≡0. Note that $\lambda_{+}$ is plotted against $10\lambda_{-}$. (b) Eigenfunctions corresponding to the large (red) and small (blue) eigenvalues, $g_{+}$ and $g_{-}$, respectively, normalized with the established fixed forcing magnitude condition, for the optimal sperm number Sp=4.7.

We find that both eigenvalues have a maximum at a single value of Sp, as illustrated in figure 3*a* (Note that, since $\lambda_{+}$ is much larger than $\lambda_{-}$, we plot $\lambda_{+}$ against $10\lambda_{-}$). We see that $\lambda_{+}$ has a maximum of $\lambda_{+}\approx 0.00170$ at Sp≈4.70, where it is approximately a hundred times larger than $\lambda_{-}$ at the same point. Furthermore, the maximum value of $\lambda_{+}$ is approximately 20 times larger than that of $\lambda_{-}$. Since $\lambda_{+}$ is much larger than $\lambda_{-}$, we set the sperm number to be Sp=4.7, for the remainder of this example. For this optimal Sp, the eigenfunctions $g_{+}$ and $g_{-}$ (for comparison) are shown in figure 3*b*.

Since $\lambda_{+}\gg \lambda_{-}$, an approximate solution may be obtained by neglecting in the analysis the smaller eigenvalue and eigenfunction. The swimming speed under continuous forcing f can then be expressed approximately as


(3.32) $$\begin{array}{lcr} & U\approx \left[{\left(\int_{0}^{1}f(s)g_{+}(s)ds\right)}^{2}-{\left(\int_{0}^{1}f(s)g_{+}(1-s)ds\right)}^{2}\right]\lambda_{+}, & \end{array}$$

or, for M discrete actuators,


(3.33) $$\begin{array}{lcr} & U\approx \left[{\left(\sum_{m=1}^{M}F_{k}g_{+}(\xi_{k})\right)}^{2}-{\left(\sum_{m=1}^{M}F_{k}g_{+}(1-\xi_{k})\right)}^{2}\right]\lambda_{+}. & \end{array}$$

These formulae represent further improvement. Since we have set Sp to take its approximate optimal value, optimizing the swimming speed over choice of f therefore produces the approximate global maximum value of U across all values of Sp, eliminating the need to vary Sp manually. These formulae also require only half as many calculations compared with equations (3.30) and (3.31).

#### (v) Swimming speed as the difference of two squares

Aiming to find the simplest mathematical method to maximize the value of U, we now define symmetric and antisymmetric functions, $g_{s}$ and $g_{a}$, respectively, to be twice the symmetric and antisymmetric components of the eigenfunction $g_{+}$:


(3.34) $$\begin{array}{lcr} & \begin{array}{rl} g_{s}(s) & =g_{+}(s)+g_{+}(1-s),\\ g_{a}(s) & =g_{+}(s)-g_{+}(1-s). \end{array} & \end{array}$$

**Figure 4.**
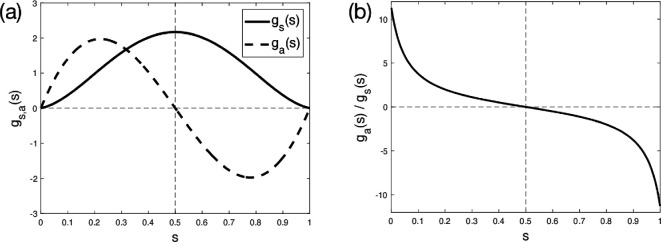
Symmetric and antisymmetric decomposition of eigenmodes. (a) Symmetric function $g_{s}$ (thick solid line) and antisymmetric function $g_{a}$ (thick dashed line); the lines g=0 and s=0.5 are shown as thin dashed lines. (b) Ratio $g_{a}(s)/g_{s}(s)$ used for optimizations.


These two functions are shown in figure 4*a*, with their ratio (to be used later) plotted in figure 4*b*. The swimming speed from equation (3.32) is then given by

(3.35) $$\begin{array}{lcr} & U\approx \left[\int_{0}^{1}f(s)g_{s}(s)ds\right]\left[\int_{0}^{1}f(s)g_{a}(s)ds\right]\lambda_{+}, & \end{array}$$

or for discrete actuators, equation (3.33) becomes


(3.36) $$\begin{array}{lcr} & U\approx \left[\sum_{m=1}^{M}F_{m}g_{s}(\xi_{m})\right]\left[\sum_{m=1}^{M}F_{m}g_{a}(\xi_{m})\right]\lambda_{+}. & \end{array}$$

In optimizing the filament, we may assume (without loss of generality) that both integrals/sums are non-negative.

#### (vi) Application to artificial swimmers with continuous forcing

An artificial swimmer is unlikely to be constrained by the fixed forcing magnitude condition given by equation (3.5); instead the limitations are more likely to be in the engineering and fabrication of the forcing on such small scales. We apply a more appropriate constraint below, but for now let us consider the simple situation where each location ξ along the filament is either passive (f(ξ)=0) or forced (f(ξ)=1). This is a reasonable parallel to the artificial microswimmer demonstrated in [25], where forcing is provided by magnetic beads (where f(ξ)≠0), potentially alternating with inert sections of filament (f(ξ)=0). Note, however, that this specific example [25] utilizes externally powered actuation, and the resultant dynamics cannot be exactly described by the model developed in this paper. Nonetheless, it is an intuitive example of the form of a microswimmer that utilizes piecewise constant forcing. By using equation (3.35), we now determine the choice of f which maximizes the swimming speed.

**Figure 5.**
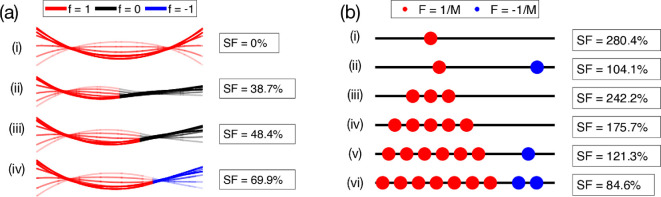
Artificial swimmers with continuous (a) and discrete (b) forcing. (a) Example artificial swimmers, with continuous, piecewise constant forcing, of progressively increasing speeds, optimized using equation 3.35. Solid lines represent the filaments at t=0, and t increases to π in progressively fading lines. Speeds have been divided by $\lambda_{+}$ to produce the speed factor (SF) as a percentage. (i) Uniformly forced filament that cannot swim due to the scallop theorem. (ii) Simple swimmer with forcing in the front half only. (iii) Swimmer with frontal forcing occupying an optimal fraction of the filament. (iv) Swimmer with optimally chosen positive and negative forcing. An animated version of these swimmers is shown in electronic supplementary material, video S1. (b) Example artificial swimmers for M=1,2,3,5,7,9 discrete actuators, each of strength 1/M, with speed factors indicated. Minimum spacing of 0.1 between actuators. An animated version of these swimmers is shown in electronic supplementary material, video S2.

Despite the temptation of using as much forcing as possible, simply setting f=1 everywhere cannot produce any swimming due to symmetry of the filament (figure 5*a(i)*), and a more detailed analysis is required. Using equation (3.35), with the functions $g_{s}$ and $g_{a}$ given in figure 4*a*, we see that, regardless of the values taken by f(ξ) for ξ>0.5, the swimming speed is always increased by setting f(ξ)=1 for all ξ≤0.5 (assuming without loss of generality that both integrals in equation (3.35) are non-negative). We immediately deduce that a possible swimmer has f(ξ)=1 for ξ≤0.5 and f(ξ)=0 for ξ>0.5 (figure 5*a(ii)*).

We can further improve this swimmer by setting f(ξ)=1 for some values of ξ>0.5. Suppose that f takes its optimal value. Then any acceptable change in f (i.e. any change which maintains f(ξ)=0 or f(ξ)=1 at each ξ) will necessarily reduce the swimming speed. Calling such a change δf(s;ξ), which takes a constant non-zero value (either 1 or −1) in a small region of width Δ around s=ξ, and is zero elsewhere, the swimming speed is perturbed by


(3.37) $$\begin{array}{lcr} & \delta U\approx \left\{\left[\int_{0}^{1}f(s)g_{s}(s)ds\right]g_{a}(\xi )+g_{s}(\xi )\left[\int_{0}^{1}f(s)g_{a}(s)ds\right]\right\}\Delta \lambda_{+}\delta f(\xi ;\xi ). & \end{array}$$

For an optimal f, we require this to be negative whenever δf takes an allowable value. Therefore, if f(ξ)=1, then δU must be negative for δf(ξ;ξ)=−1, while if f(ξ)=0, then δU must be negative for δf(ξ;ξ)=1. We deduce that


(3.38)ga(ξ)(∫01f(s)gs(s) ds)+gs(ξ)(∫01f(s)ga(s) ds)≥0          when  f(ξ)=1,(3.39)ga(ξ)(∫01f(s)gs(s) ds)+gs(ξ)(∫01f(s)ga(s) ds)≤0          when  f(ξ)=0.

By noting that $g_{s}$ is positive, we can write these inequalities as


(3.40)∫01f(s)ga(s) ds∫01f(s)gs(s) ds+ga(ξ)gs(ξ)≥0          when  f(ξ)=1,(3.41)∫01f(s)ga(s) ds∫01f(s)gs(s) ds+ga(ξ)gs(ξ)≤0          when  f(ξ)=0.

As shown in figure 4*b*, the ratio $g_{a}/g_{s}$ is an antisymmetric, strictly decreasing function of ξ. Therefore, assuming that f does not take the same constant value for all ξ, an optimal f that satisfies these inequalities must have a corresponding point $\xi_{1}^{*}$ such that


(3.42) $$\begin{array}{lcr} & \frac{\int_{0}^{1}f(s)g_{a}(s)ds}{\int_{0}^{1}f(s)g_{s}(s)ds}=-\frac{g_{a}(\xi_{1}^{*})}{g_{s}(\xi_{1}^{*})}, & \end{array}$$

with f(ξ)=1 for $\xi <\xi_{1}^{*}$ and f(ξ)=0 for $\xi >\xi_{1}^{*}$. Hence this implicit equation can be simplified as


(3.43) $$\begin{array}{lcr} & \frac{\int_{0}^{\xi_{1}^{*}}g_{a}(s)ds}{\int_{0}^{\xi_{1}^{*}}g_{s}(s)ds}=-\frac{g_{a}(\xi_{1}^{*})}{g_{s}(\xi_{1}^{*})}. & \end{array}$$

We can solve equation (3.43) numerically to identify $\xi_{1}^{*}=0.625$, with the corresponding swimmer illustrated in figure 5*a(iii)*.

This procedure can easily be adapted to consider a somewhat more advanced swimmer where f can be 1, 0 or −1. Following the same logic we obtain


(3.44)∫01f(s)ga(s) ds∫01f(s)gs(s) ds+ga(ξ)gs(ξ)≥0          when  f(ξ)=1,(3.45)∫01f(s)ga(s) ds∫01f(s)gs(s) ds+ga(ξ)gs(ξ)=0          when  f(ξ)=0,(3.46)∫01f(s)ga(s) ds∫01f(s)gs(s) ds+ga(ξ)gs(ξ)≤0          when  f(ξ)=−1.

Once again, there is therefore a point $\xi_{2}^{*}$ for which f(ξ)=1 for $\xi <\xi_{2}^{*}$ and f(ξ)=−1 for $\xi >\xi_{2}^{*}$. There can be no region where f=0 because that would require equation (3.45) to be satisfied within said region, which is impossible for the strictly decreasing ratio $g_{a}/g_{s}$. Then $\xi_{2}^{*}$ is now given by


(3.47) $$\begin{array}{lcr} & \frac{\int_{0}^{\xi_{2}^{*}}g_{a}(s)ds-\int_{\xi_{2}^{*}}^{1}g_{a}(s)ds}{\int_{0}^{\xi_{2}^{*}}g_{s}(s)ds-\int_{\xi_{2}^{*}}^{1}g_{a}(s)ds}=-\frac{g_{a}(\xi_{2}^{*})}{g_{s}(\xi_{2}^{*})}. & \end{array}$$

The implicit equation in equation (3.47) can be solved to find $\xi_{2}^{*}=0.701$, resulting in the swimmer shown in figure 5*a(iv)*.

The four artificial swimmers shown in figure 5*a* have progressively increasing speeds, as indicated in insets and quantified as speed fraction (SF), defined as the dimensionless speed divided by the eigenvalue $\lambda_{+}$ and interpreted as the fraction of the swimming speed U that would be achieved by setting the forcing function f to be the eigenfunction $g_{+}$. We see in particular that this final artificial swimmer can achieve a maximum of 69.9% of its optimal speed, as defined by setting $f=g_{+}$. Animated versions of the swimmers of figure 5*a* are available in electronic supplementary material, video S1.

#### (vii) Piecewise constant forcing is optimized in the limit of single-point forcing

An optimal SF of 69.9% (figure 5*a(iv)*) is fairly respectable, and an artificial swimmer with this piecewise constant forcing would be a comparably fast swimmer with one that uses eigenfunction forcing. It is notable that the swimmer in figure 5*a(iv)* has a swimming speed that is less than double the swimming speed of the swimmer of figure 5*a(ii)*, despite having twice as much total forcing. Indeed, uniformly doubling the forcing magnitude (from f(ξ) to 2f(ξ)) of any given swimmer would quadruple the swimming speed, which makes an improvement of less than double disappointing, and this motivates a new forcing constraint. An artificial swimmer is likely to be limited by the engineering involved in its fabrication, and this is likely to manifest as a total forcing magnitude constraint of the form


(3.48) $$\begin{array}{lcr} & \int_{0}^{1}|f(\xi )|d\xi =1. & \end{array}$$

A specific example of this would be the artificial millimetre-scale swimmer powered by cardiomyocytes (heart muscle cells) studied experimentally in [30], where each cardiomyocyte generates a fixed forcing moment. In that case, the most likely limitation is the number of muscle cells that can be cultured onto the filament, corresponding to the new forcing magnitude constraint in equation (3.48). Note that, under this constraint, $g_{+}$ has a total forcing magnitude of approximately 0.84, rather than 1. Eigenfunction forcing under this new total forcing magnitude constraint (i.e. $f=g_{+}/0.84$) will therefore produce a speed factor of 142.0%.

Returning to figure 5*a*, we observe that a swimmer that has f(ξ)=2 for ξ≤0.5 and f(ξ)=0 for ξ>0.5 (i.e. as in figure 5*a(ii)* but with doubled forcing and hence quadrupled speed) would swim more than twice as fast as one with the more distributed forcing |f(ξ)|=1 for all ξ (figure 5*a(iv)*), for the same total forcing magnitude as defined by equation (3.48). This suggests that distributing the forcing along a greater length is, perhaps counterintuitively, detrimental to swimming. This is in contrast to the biological situation of spermatozoa, which use distributed forcing along the entire axoneme [9–12].

In fact, as we are now going to show, in this new constraint swimming is optimized by taking the limit of single-point actuation; this will turn out to present up to fourfold improvement compared with the swimmer of figure 5*a(iv)*, while still satisfying equation 3.48.

From previous swimmers illustrated in figure 5*a*, it is apparent from the signs of $g_{s}$ and $g_{a}$ (figure 4*a*) and of their ratio $g_{a}/g_{s}$ (figure 4*b*), that positive forcing (f≥0) should exist in 0≤ξ≤0.5, and extending some way past 0.5, while negative forcing (f≤0) should occupy the remainder of the filament.

In the range 0≤ξ≤0.221, both $g_{s}$ and $g_{a}$ are increasing (figure 4*a*) and so any positive forcing in this range should be moved right if possible. Conversely, for 0.5≤ξ≤0.779, both $g_{s}$ and $g_{a}$ are decreasing and any positive forcing should be moved to the left. In the region 0.221≤ξ≤0.5, it is easily shown that $g_{s}$ and $g_{a}$ are both concave functions, and thus contracting any positive forcing (e.g. changing f(ξ)=F for $\xi_{1}\leq \xi \leq \xi_{2}$ into $f(\xi )=F\times \left(\xi_{2}-\xi_{1}\right)/\left(\xi_{2}-\xi_{1}-2\epsilon \right)$ for $\xi_{1}+\epsilon \leq \xi \leq \xi_{2}-\epsilon$) will necessarily increase the swimming speed, since it would be increasing both terms in equation (3.35). From these observations, we deduce that the benefit to the swimming speed contributed by the positive forcing can be improved by contracting it, ideally to the limit of single-point actuation at some position in the interval 0.221≤ξ≤0.5.

Conversely, any negative forcing in the range 0.5≤ξ≤0.779 should be moved rightwards; in particular, this implies that all negative forcing should exist entirely to the right of any positive forcing. While $g_{s}$ is slightly concave in a small sub-region at the start of 0.779≤ξ≤1, it is convex in most of the region, with $g_{a}$ being convex in the entire region. Overall, swimming speed is therefore essentially increased by a contraction of the negative forcing in this region. Therefore we deduce that negative forcing should again be contracted as much as possible, ideally to the limit of single-point actuation at some point in the range 0.779≤ξ≤1, to produce near-optimal swimming speed.

#### (viii) Optimizing swimmers under discrete actuation

Having shown that piecewise constant forcing is optimized in the limit of single-point forcing, we now investigate numerically the optimal configurations, directly applying the results to the biohybrid swimmer of [30] shortly. Starting with the case of a single-point positive forcing, $f(\xi )=\delta (\xi -\xi_{1})$, and no negative forcing, we find that swimming speed is maximized by placing the actuator at $\xi_{1}=0.309$, achieving a far greater swimming SF of 280.4% (see swimmer illustrated in figure 5*b(i)*). It is perhaps surprising that concentrating all the forcing in a single location can produce a far greater swimming speed compared even to eigenfunction forcing under the same fixed forcing magnitude constraint (equation (3.48)), with the speed factor almost doubling. In addition, suppose we instead want to use one positive and one negative forcing actuator of equal strength, 1/2. We find these should be placed at $\xi_{1}=0.357$ and $\xi_{2}=0.902$, respectively, though this only achieves an SF of 104.1% (figure 5*b(ii)*).

By varying over all possible allocations of forcing magnitudes between these two actuators, we find that just having a single, positive actuator of strength 1 at $\xi_{1}=0.309$, and no negative actuator (i.e. the swimmer of figure 5*b(i)*), is optimal among all possible discrete actuations. Across the entire suite of forcing functions, the optimal swimming speed subject to the new constraint equation (3.48) is thus achieved using this single-point actuator.

From a practical standpoint, there may be situations in which an artificial swimmer cannot be constructed with all of its forcing applied to one location. For example, in the cardiomyocytes-powered swimmer from [30], the magnitude of the forcing that can be applied at a particular location is limited by the contractile force of the cardiomyocytes that power the filament. Since the filament has limited space and one cannot simply place arbitrarily many of these cardiomyocytes at $\xi_{1}=0.309$, the forcing would probably need to be distributed over some length, using some number M of single-point actuators, possibly limited by some minimum spacing requirement between the actuators.

If the locations of the actuators were pre-determined (e.g. $\xi_{m}=(2m-1)/2M$), and we are free to choose the forcing strengths ($F_{m}=1/M$, 0 or −1/M), then the results in figure 5*a* serve as an excellent guide when M is large. In addition, brute force computations are feasible when M is small, since there are only $3^{M}$ configurations and their number can be further reduced using the logic we have considered above (e.g. noting that actuators in ξ≤0.5 should have positive forcing).

On the other hand, the actuator locations may not be pre-defined, and we may instead be free to choose the optimal actuator location and forcing direction for a given number of actuators. As we have argued above, all actuators with the same forcing should be placed as close together as possible. We may assume (by symmetry) that at least half of the actuators have positive forcing, giving at most M/2 configurations for the number of actuators of each sign. For each of these, we are left with just two continuous parameters to optimize: the locations $\xi_{1}$ and $\xi_{2}$ of the centre of each group of actuators. In figure 5*b(iii–vi)*, we illustrate the results obtained for M=3,5,7,9*,* assuming a minimum actuator separation of 0.1. As expected, we see that distributing the forcing over multiple actuators progressively decreases the swimming speed. For larger values of M, the inclusion of negative forcing actuators becomes necessary to achieve optimal swimming speed (figure 5*b(v,vi)*). Animated versions of the swimmers of figure 5*b* are available in electronic supplementary material, video S2.

#### (ix) Application to the biohybrid swimmer of Ref. [30]

The results of this paper, in particular those of figure 5*b*, can be applied to perform optimizations based on various constraints such as actuator separation and direction. A good example of such a swimmer is the approximately 2mm long biohybrid swimmer produced in [30]. This swimmer (which, in the experiments, possessed a passive head) was actuated by cardiomyocytes, cultured onto the flagellum near the point of attachment with the head. By applying our model, we can identify the optimal configuration for a headless swimmer, and compare our results to the numerical results obtained in [30] in the limit of a vanishing head. Simply applying the same value of Sp (4.19) and the same actuator location (around ξ=0.25) used in their experiments, we calculate a dimensional swimming speed of approximately 1.3μm s^−1^, in good agreement with their numerical results (1 to 2μm s^−1^). This agreement is despite the fact that the dimensionless moment forcing has a magnitude of approximately 4.5, suggesting our model remains reasonably accurate even for nonlinear actuation. Furthermore, the cardiomyocyte contractions were not simple sinusoidal functions of time, and higher temporal modes were present, though this is easily resolved since the temporal modes decouple and the overall dimensional swimming is simply the sum of the dimensional swimming speeds corresponding to each temporal mode. Modifying Sp (for example, by elongating the swimmer) and ξ to take their optimal values of 4.7 and 0.309, respectively (see figure 5*b(i)*), can increase this dimensional swimming speed, measured in swimmer lengths per second, by approximately 50%. Importantly, this is an immediate result requiring no additional analysis or computation.

## 4. Conclusion

The dynamics of slender filaments is relevant not only for modelling biological microorganisms such as spermatozoa, but also for the design and fabrication of artificial swimmers. By revisiting and linearizing classical elastohydrodynamic theory, we have derived simple expressions for the shape of the filament (equation (2.29)) in terms of Green’s function (appendix A) and for the swimming speed (equation (2.35)) in terms of the swimming speed function (equation (2.36)). In particular, the swimming speed may be evaluated, for a given forcing, without the need to explicitly calculate the shape of the filament, in an $O\left(N^{2}\right)$ process, where N is the number of points used to perform the numerical integration in equation (2.35). However, while useful for the quick and easy evaluation of the swimming speed, this process is not conducive to optimizations over choices of forcing functions.

The real symmetric nature of the swimming speed function suggests a modal approach, and we next numerically identified its eigenfunctions and eigenvalues by discretizing the swimming speed function as a large symmetric matrix. We further revealed via calculus of variations that these eigenfunctions provide optimal forcing functions for swimming, assuming a fixed forcing magnitude constraint (equation (3.5)). These eigenfunctions and eigenvalues provide an alternative method by which to calculate the swimming speed (equation (3.2)), and in particular most of the modes may be neglected by virtue of small eigenvalues. We have found that, in a variety of situations, only a small number of eigenmodes need be retained to produce accurate results (figure 2), thereby reducing the computational complexity in evaluating the swimming speed to an O(N) problem, or even an O(1) problem if the forcing function is initially constructed in terms of the modal basis.

Furthermore, this modal approach allows for optimizations of the forcing function, such as by approximating it as a sum of the dominant modes. In the particular situation of monophasic forcing, the eigenfunctions and eigenvalues may be calculated analytically, and in particular only four of the infinitely many eigenvalues are non-zero. These consist of two symmetric pairs, one of which is dominated by the other at optimal Sp (figure 3). By neglecting the lesser eigenvalue, we have revealed a wide range of analyses that allow for the optimal design of an artificial swimmer, subject to various constraints (figure 5), in particular producing effective swimmers that utilize piecewise constant forcing or single-point actuation, relevant to previously demonstrated artificial swimmers [25,30].

It is clear that the slender filaments considered are but a simplified model system. Real swimmers, both biological and artificial, typically carry a body. Not only does this allow for the transport of a payload, but the presence of a body can also be advantageous for producing increased swimming speed [30]. Furthermore, we have considered here only two-dimensional filament motion, while some spermatozoa can exhibit three-dimensional (often helical) motion [38–40]. Finally, our assumption of small (ϵ≪1) disturbances will inevitably lead to inaccuracies when modelling real swimmers with O(1) disturbances, though previous investigations have shown remarkable agreement even in such situations [18]. Despite these simplifications, we have obtained results which are quantitatively similar to those observed in spermatozoa swimming through *in vitro* fertilization medium (figure 2). Potential adaptations and improvements to this work include generalizations of the mathematical approach, such as considering different variational constraints which may produce new and interesting optimizations. Future work could also consider generalizations of the swimmer design and behaviour, such as through the inclusion of a head, as in spermatozoa and the aforementioned artificial swimmers, which have an effective head either in the form of a payload [25], or a designed head that improves swimming [30].

We hope that this work will be helpful in the design and optimization of artificial swimmers, and in aiding the ongoing understanding of the motion of spermatozoa and other microorganisms, with potential applications to fertility science, micro-engineering and general medical applications.

## Data Availability

MATLAB code used to generate figures has been made publicly available at [41]. Supplementary material is available online [42].

## Appendix A. Green’s function for the hyperdiffusion equation

The Green’s function G(s;ξ) used in equation (2.29) is the solution to the equation

(A 1)
$$\begin{array}{lcr} & Sp^{4}iG+G^{\prime \prime \prime \prime }=\delta (s-\xi ). & \end{array}$$

Note this corresponds to a single point actuator forcing located at position ξ along the filament. To enforce the zero force and moment boundary conditions on the filament, we require $G=G_{s}=0$ at s=0 and s=1. In addition, as standard for a Green’s function, G, $G^{\prime }$ and $G^{\prime \prime }$ must be continuous at s=ξ, with $G^{\prime \prime \prime }$ jumping by a value of 1. The solution for G is given by

(A 2)
$$\begin{array}{lcr} & G=H(s-\xi )G_{+}+H(\xi -s)G_{-}, & \end{array}$$

where

(A 3)
$$G_{-}(s)=A_{-}e^{Sp\eta s}+B_{-}e^{Sp\eta is}+C_{-}e^{-Sp\eta s}+D_{-}e^{-Sp\eta is},$$

(A 4)
$$G_{+}(s)=A_{+}e^{Sp\eta s}+B_{+}e^{Sp\eta is}+C_{+}e^{-Sp\eta s}+D_{+}e^{-Sp\eta is},$$

with $\eta =e^{-\pi i/8}$. The coefficients are determined by the boundary and jump conditions, via the matrix equation

(A 5)
$$\begin{array}{lcr} & \begin{array}{r} \left[\begin{array}{cccccccc} 1 & 1 & 1 & 1 & 0 & 0 & 0 & 0\\ 1 & i & -1 & -i & 0 & 0 & 0 & 0\\ 0 & 0 & 0 & 0 & e^{Sp\eta } & e^{Sp\eta i} & e^{-Sp\eta } & e^{-Sp\eta i}\\ 0 & 0 & 0 & 0 & e^{Sp\eta } & ie^{Sp\eta i} & -e^{-Sp\eta } & -ie^{-Sp\eta i}\\ -e^{Sp\eta \xi } & -e^{Sp\eta i\xi } & -e^{-Sp\eta \xi } & -e^{-Sp\eta i\xi } & e^{Sp\eta \xi } & e^{Sp\eta i\xi } & e^{-Sp\eta \xi } & e^{-Sp\eta i\xi }\\ -e^{Sp\eta \xi } & -ie^{Sp\eta i\xi } & e^{-Sp\eta \xi } & ie^{-Sp\eta i\xi } & e^{Sp\eta \xi } & ie^{Sp\eta i\xi } & -e^{-Sp\eta \xi } & -ie^{-Sp\eta i\xi }\\ -e^{Sp\eta \xi } & e^{Sp\eta i\xi } & -e^{-Sp\eta \xi } & e^{-Sp\eta i\xi } & e^{Sp\eta \xi } & -e^{Sp\eta i\xi } & e^{-Sp\eta \xi } & -e^{-Sp\eta i\xi }\\ -e^{Sp\eta \xi } & ie^{Sp\eta i\xi } & e^{-Sp\eta \xi } & -ie^{-Sp\eta i\xi } & e^{Sp\eta \xi } & -ie^{Sp\eta i\xi } & -e^{-Sp\eta \xi } & ie^{-Sp\eta i\xi } \end{array}\right]\left[\begin{array}{c} A_{-}\\ B_{-}\\ C_{-}\\ D_{-}\\ A_{+}\\ B_{+}\\ C_{+}\\ D_{+} \end{array}\right]=\left[\begin{array}{c} 0\\ 0\\ 0\\ 0\\ 0\\ 0\\ 0\\ \frac{1}{Sp^{3}\eta^{3}} \end{array}\right]. \end{array} & \end{array}$$

We have therefore solved the hyperdiffusion equation for the Green’s function G.

## Appendix B. Derivation of equation (2.32)

Recalling that

(B 1)
$$\begin{array}{lcr} & U=-2\int_{0}^{1}\left\langle \Psi_{sss}\Psi_{sst}\right\rangle ds, & \end{array}$$

we derive here equation (2.32) for U, for general moment forcing $m^{\left(1\right)}(s,t)=R\left[f(s)e^{-i\phi (s)}e^{it}\right]$ and corresponding solution $\Psi (s,t)=R\left[e^{it}\int_{0}^{1}G(s;\xi )f(\xi )e^{-i\phi (\xi )}\;d\xi \right]$. We begin by substituting the solution for Ψ, moving the time-average inside the integrals:

(B 2)
$$U=-2\int_{s=0}^{1}\int_{\xi_{1}=0}^{1}\int_{\xi_{2}=0}^{1}\left\langle R\left[e^{it}G^{\prime \prime \prime }(s;\xi_{1})f(\xi_{1})e^{-i\phi (\xi_{1})}\right]R\left[ie^{it}G^{\prime \prime }(s;\xi_{2})f(\xi_{2})e^{-i\phi (\xi_{2})}\right]\right\rangle \;d\xi_{2}d\xi_{1}\;ds.$$

Next we evaluate the time-averages to find

(B 3)
$$\begin{array}{rl} U=\int_{s=0}^{1}\int_{\xi_{1}=0}^{1}\int_{\xi_{2}=0}^{1} & \{-I\left[G^{\prime \prime \prime }(s;\xi_{1})f(\xi_{1})e^{-i\phi (\xi_{1})}\right]R\left[G^{\prime \prime }(s;\xi_{2})f(\xi_{2})e^{-i\phi (\xi_{2})}\right]\\ & \;\;\;+R\left[G^{\prime \prime \prime }(s;\xi_{1})f(\xi_{1})e^{-i\phi (\xi_{1})}\right]I\left[G^{\prime \prime }(s;\xi_{2})f(\xi_{2})e^{-i\phi (\xi_{2})}\right]\}\;d\xi_{2}d\xi_{1}ds. \end{array}$$

We integrate by parts with respect to s, recalling that $G^{\prime }(0;\xi )=G^{\prime }(1;\xi )=0$,

(B 4)
$$\begin{array}{rl} U=\int_{s=0}^{1}\int_{\xi_{1}=0}^{1}\int_{\xi_{2}=0}^{1} & \{I\left[G^{⁗}(s;\xi_{1})f(\xi_{1})e^{-i\phi (\xi_{1})}\right]R\left[G^{\prime }(s;\xi_{2})f(\xi_{2})e^{-i\phi (\xi_{2})}\right]\\ & -R\left[G^{⁗}(s;\xi_{1})f(\xi_{1})e^{-i\phi (\xi_{1})}\right]I\left[G^{\prime }(s;\xi_{2})f(\xi_{2})e^{-i\phi (\xi_{2})}\right]\}\;d\xi_{2}d\xi_{1}ds. \end{array}$$

We now recall the governing equation for the Green’s function, $Sp^{4}iG+G^{\prime \prime \prime \prime }=\delta (s-\xi )$, giving

(B 5)
$$\begin{array}{rl} U=\int_{s=0}^{1}\int_{\xi_{1}=0}^{1}\int_{\xi_{2}=0}^{1} & \{I\left[\left(\delta (s-\xi_{1})-Sp^{4}iG(s;\xi_{1})\right)f(\xi_{1})e^{-i\phi (\xi_{1})}\right]R\left[G^{\prime }(s;\xi_{2})f(\xi_{2})e^{-i\phi (\xi_{2})}\right]\\ & -R\left[\left(\delta (s-\xi_{1})-Sp^{4}iG(s;\xi_{1})\right)f(\xi_{1})e^{-i\phi (\xi_{1})}\right]I\left[G^{\prime }(s;\xi_{2})f(\xi_{2})e^{-i\phi (\xi_{2})}\right]\}\;d\xi_{2}d\xi_{1}ds. \end{array}$$

Using integration by parts with respect to s, recalling that G(0;ξ)=G(1;ξ)=0, we note that

(B 6)
$$\begin{array}{rl} I(\xi_{1},\xi_{2}) & =\int_{s=0}^{1}R\left[G(s;\xi_{1})f(\xi_{1})e^{-i\phi (\xi_{1})}\right]R\left[G^{\prime }(s;\xi_{2})f(\xi_{2})e^{-i\phi (\xi_{2})}\right]\;ds\\ & =-\int_{s=0}^{1}R\left[G^{\prime }(s;\xi_{1})f(\xi_{1})e^{-i\phi (\xi_{1})}\right]R\left[G(s;\xi_{2})f(\xi_{2})e^{-i\phi (\xi_{2})}\right]\;ds=-I(\xi_{2},\xi_{1}). \end{array}$$

Therefore

(B 7)
$$\begin{array}{lcr} & \int_{\xi_{1}=0}^{1}\int_{\xi_{2}=0}^{1}I(\xi_{1},\xi_{2})d\xi_{2}d\xi_{1}=\int_{\xi_{1}=0}^{1}\int_{\xi_{2}=0}^{1}-I(\xi_{2},\xi_{1})d\xi_{2}d\xi_{1}=\int_{\xi_{2}=0}^{1}\int_{\xi_{1}=0}^{1}-I(\xi_{1},\xi_{2})d\xi_{1}d\xi_{2}, & \end{array}$$

and so is zero. The same argument holds for $I(\xi_{1},\xi_{2})$ defined using imaginary parts rather than real parts. Therefore the equation for U simplifies to

(B 8)
$$\begin{array}{lcr} & \begin{array}{rl} U=\int_{s=0}^{1}\int_{\xi_{1}=0}^{1}\int_{\xi_{2}=0}^{1} & \{ℑ\left[\delta (s-\xi_{1})f(\xi_{1})e^{-i\phi (\xi_{1})}\right]ℜ\left[G^{\prime }(s;\xi_{2})f(\xi_{2})e^{-i\phi (\xi_{2})}\right]\\ & -ℜ\left[\delta (s-\xi_{1})f(\xi_{1})e^{-i\phi (\xi_{1})}\right]ℑ\left[G^{\prime }(s;\xi_{2})f(\xi_{2})e^{-i\phi (\xi_{2})}\right]\}d\xi_{2}d\xi_{1}ds. \end{array} & \end{array}$$

The δ-functions are real and so

(B 9)
$$\begin{array}{lcr} & \begin{array}{rl} U=\int_{s=0}^{1}\int_{\xi_{1}=0}^{1}\int_{\xi_{2}=0}^{1} & \{\delta (s-\xi_{1})ℑ\left[f(\xi_{1})e^{-i\phi (\xi_{1})}\right]ℜ\left[G^{\prime }(s;\xi_{2})f(\xi_{2})e^{-i\phi (\xi_{2})}\right]\\ & -\delta (s-\xi_{1})ℜ\left[f(\xi_{1})e^{-i\phi (\xi_{1})}\right]ℑ\left[G^{\prime }(s;\xi_{2})f(\xi_{2})e^{-i\phi (\xi_{2})}\right]\}d\xi_{2}d\xi_{1}ds, \end{array} & \end{array}$$

and we now evaluate the integral in s to obtain

(B 10)
$$\begin{array}{lcr} & \begin{array}{rl} U=\int_{\xi_{1}=0}^{1}\int_{\xi_{2}=0}^{1} & \{ℑ\left[f(\xi_{1})e^{-i\phi (\xi_{1})}\right]ℜ\left[G^{\prime }(\xi_{1};\xi_{2})f(\xi_{2})e^{-i\phi (\xi_{2})}\right]\\ & -ℜ\left[f(\xi_{1})e^{-i\phi (\xi_{1})}\right]ℑ\left[G^{\prime }(\xi_{1};\xi_{2})f(\xi_{2})e^{-i\phi (\xi_{2})}\right]\}d\xi_{2}d\xi_{1}. \end{array} & \end{array}$$

Recalling that f is real gives

(B 11)
$$\begin{array}{lcr} & U=\int_{\xi_{1}=0}^{1}\int_{\xi_{2}=0}^{1}f(\xi_{1})\left(ℑ\left[e^{-i\phi (\xi_{1})}\right]ℜ\left[G^{\prime }(\xi_{1};\xi_{2})e^{-i\phi (\xi_{2})}\right]-ℜ\left[e^{-i\phi (\xi_{1})}\right]ℑ\left[G^{\prime }(\xi_{1};\xi_{2})e^{-i\phi (\xi_{2})}\right]\right)f(\xi_{2})d\xi_{2}d\xi_{1}. & \end{array}$$

And by re-combining terms, we obtain

(B 12)
$$\begin{array}{lcr} & U=-\int_{\xi_{1}=0}^{1}\int_{\xi_{2}=0}^{1}f(\xi_{1})ℑ\left[G^{\prime }(\xi_{1};\xi_{2})e^{i(\phi (\xi_{1})-\phi (\xi_{2}))}\right]f(\xi_{2})d\xi_{2}d\xi_{1}, & \end{array}$$

as promised.

## Appendix C. Standard eigenfunction calculation for monophasic forcing

The method presented in the main text allows for the concise calculation of the eigenfunctions and eigenvalues for the case ϕ≡0. We present here a more standard method that has potential to be generalized to different situations, at the cost of being more cumbersome and producing an 8×8 system with four zero eigenvalues.

Suppose $f(\xi_{1})=ℜ\left[e^{k\xi_{1}}\right]=\frac{1}{2}\left(e^{k\xi_{1}}+e^{k^{*}\xi_{1}}\right)$, where k is one of the four values such that this is a natural mode; $k^{4}=-Sp^{4}i$. Note that $k^{*}$ does not correspond to a natural mode. Then we can solve for $I_{1}$ and $I_{2}$ as

(C1)I1(1,k)(ξ1)=ξ1ekξ18k3+ek∗ξ14Sp4i+A1(1,k)eSpηξ1+B1(1,k)eSpηiξ1+C1(1,k)e−Spηξ1+D1(1,k)e−Spηiξ1,(C2)I2(1,k)(ξ1)=ξ1ekξ18k2+k∗ek∗ξ14Sp4i+A2(1,k)eSpηξ1+B2(1,k)eSpηiξ1+C2(1,k)e−Spηξ1+D2(1,k)e−Spηiξ1.

Alternatively, if $f(\xi_{1})=ℜ\left[ie^{k\xi_{1}}\right]$, then $I_{1}$ and $I_{2}$ are given by

(C3)I1(i,k)(ξ1)=iξ1ekξ18k3−ek∗ξ14Sp4+A1(i,k)eSpηξ1+B1(i,k)eSpηiξ1+C1(i,k)e−Spηξ1+D1(i,k)e−Spηiξ1,(C4)I2(i,k)(ξ1)=iξ1ekξ18k2−k∗ek∗ξ14Sp4+A2(i,k)eSpηξ1+B2(i,k)eSpηiξ1+C2(i,k)e−Spηξ1+D2(i,k)e−Spηiξ1.

The 64 coefficients A,B,C,D can easily be calculated, for a particular Sp, according to the boundary conditions $I(0)=I(1)=I^{\prime }(0)=I^{\prime }(1)=0$. From these, we can define, for b=1 or b=i, and a particular value of k,

(C 5)
$$\begin{array}{lcr} & I_{3}^{\left(b,k\right)}=\frac{1}{2}i\left({\left(I_{1}^{\left(b,k\right)}\right)}^{\prime }-I_{2}^{\left(b,k\right)}\right), & \end{array}$$

which, defining new coefficients,

(C 6)
$$\begin{array}{lcr} & \begin{array}{rl} A_{3}^{\left(b,k\right)} & =\frac{1}{2}i\left(Sp\eta A_{1}^{\left(b,k\right)}-A_{2}^{\left(b,k\right)}\right),\\ B_{3}^{\left(b,k\right)} & =\frac{1}{2}i\left(Sp\eta iB_{1}^{\left(b,k\right)}-B_{2}^{\left(b,k\right)}\right),\\ C_{3}^{\left(b,k\right)} & =\frac{1}{2}i\left(-Sp\eta C_{1}^{\left(b,k\right)}-C_{2}^{\left(b,k\right)}\right),\\ D_{3}^{\left(b,k\right)} & =\frac{1}{2}i\left(-Sp\eta iD_{1}^{\left(b,k\right)}-D_{2}^{\left(b,k\right)}\right), \end{array} & \end{array}$$

can be evaluated as

(C 7)
$$\begin{array}{lcr} & I_{3}^{\left(b,k\right)}(\xi_{1})=A_{3}^{\left(b,k\right)}e^{Sp\eta \xi_{1}}+B_{3}^{\left(b,k\right)}e^{Sp\eta i\xi_{1}}+C_{3}^{\left(b,k\right)}e^{-Sp\eta \xi_{1}}+D_{3}^{\left(b,k\right)}e^{-Sp\eta i\xi_{1}}. & \end{array}$$

Importantly, the 32 coefficients can be evaluated easily for any given Sp. Therefore, letting general $f(\xi_{1})$ be given by the sum of its eight possible terms,

(C 8)
$$\begin{array}{lcr} & f(\xi_{1})=\sum_{\begin{array}{c} b=1,i\\ k^{4}=-Sp^{4}i \end{array}}E^{\left(b,k\right)}ℜ\left[ae^{k\xi_{1}}\right], & \end{array}$$

where each $E^{\left(b,k\right)}$ is real, we obtain an overall $I_{3}$ given by

(C 9)
$$\begin{array}{lcr} & I_{3}(\xi_{1})=\sum_{\begin{array}{c} b=1,i\\ k^{4}=-Sp^{4}i \end{array}}E^{\left(b,k\right)}\left(A_{3}^{\left(b,k\right)}e^{Sp\eta \xi_{1}}+B_{3}^{\left(b,k\right)}e^{Sp\eta i\xi_{1}}+C_{3}^{\left(b,k\right)}e^{-Sp\eta \xi_{1}}+D_{3}^{\left(b,k\right)}e^{-Sp\eta i\xi_{1}}\right). & \end{array}$$

The eigenvalue problem can then be written as

(C 10)
$$\begin{array}{lcr} & \begin{array}{rl} & \lambda \sum_{\begin{array}{c} b=1,i\\ k^{4}=-Sp^{4}i \end{array}}E^{\left(b,k\right)}ℜ\left[be^{k\xi_{1}}\right]\\ = & \sum_{\begin{array}{c} b=1,i\\ k^{4}=-Sp^{4}i \end{array}}E^{\left(b,k\right)}ℜ\left[A_{3}^{\left(b,k\right)}e^{Sp\eta \xi_{1}}+B_{3}^{\left(b,k\right)}e^{Sp\eta i\xi_{1}}+C_{3}^{\left(b,k\right)}e^{-Sp\eta \xi_{1}}+D_{3}^{\left(b,k\right)}e^{-Sp\eta i\xi_{1}}\right]. \end{array} & \end{array}$$

Equating each of the eight modes gives an 8×8 eigenvalue problem, for a vector v of the coefficients $E^{\left(b,k\right)}$, and matrix $M_{eig}$,

(C 11)
$$\begin{array}{lcr} & \lambda v=M_{eig}v. & \end{array}$$

The easiest way to write this explicitly is to define the *vectorization* of a set of coefficients by

(C 12)
$$\begin{array}{lcr} & vec(F)=\left[\begin{array}{c} F^{\left(1,Sp\eta \right)}F^{\left(1,Sp\eta i\right)}F^{\left(1,-Sp\eta \right)}F^{\left(1,-Sp\eta i\right)}F^{\left(i,Sp\eta \right)}F^{\left(i,Sp\eta i\right)}F^{\left(i,-Sp\eta \right)}F^{\left(i,-Sp\eta i\right)} \end{array}\right]. & \end{array}$$

Then

(C 13)
$$\begin{array}{lcr} & v={vec(E)}^{⊺}, & \end{array}$$

and

(C 14)
$$\begin{array}{lcr} & M_{eig}=\left[\begin{array}{c} ℜ\left[vec(A_{3})\right]\\ ℜ\left[vec(B_{3})\right]\\ ℜ\left[vec(C_{3})\right]\\ ℜ\left[vec(D_{3})\right]\\ ℑ\left[vec(A_{3})\right]\\ ℑ\left[vec(B_{3})\right]\\ ℑ\left[vec(C_{3})\right]\\ ℑ\left[vec(D_{3})\right] \end{array}\right]. & \end{array}$$

As mentioned previously, four of the eight resultant eigenvalues are zero, leaving four non-zero eigenvalues, as promised.
